# Time-dependent efficacy of seven pharmacological regimens for ulcer healing and delayed bleeding prevention after gastric endoscopic submucosal dissection: A frequentist network meta-analysis

**DOI:** 10.1371/journal.pone.0362119

**Published:** 2026-11-10

**Authors:** Linqiang Li, Xiaowei Zhu, Hengda Hu

**Affiliations:** 1 Department of Gastroenterology, Tongxiang Hospital of Traditional Chinese Medicine, Tongxiang City, Zhejiang Province, China; 2 Department of Urology, Jinshan District Central Hospital affiliated to Shanghai University of Medicine & Health Sciences, Jinshan Branch of Shanghai Sixth People’s Hospital, Jinshan District, Shanghai, China; Shanghai Jiao Tong University Medical School Affiliated Ruijin Hospital, CHINA

## Abstract

**Background:**

Gastric ESD induces large mucosal defects with potential delayed complications, including bleeding and perforation. The comparative efficacy of pharmacological regimens—PPI, P-CAB, H2RA, MP, and their combinations—remains unclear.

**Methods:**

We conducted a frequentist network meta-analysis of 31 randomized controlled trials evaluating seven pharmacological strategies for post-ESD ulcer healing and delayed bleeding prevention. Outcomes were assessed at 4 and 8 weeks for mucosal healing and ulcer reduction, and across the treatment course for delayed bleeding/perforation. Odds ratios (ORs), mean differences (MDs), and P-scores were calculated. Heterogeneity, inconsistency, sensitivity, and publication bias were assessed. Transitivity was evaluated qualitatively, and certainty of evidence was rated using the GRADE framework.

**Results:**

At 4 weeks, the point estimate for P-CAB monotherapy favored PPI (OR=1.61, 95%CI 0.96–2.68, P = 0.069), but the confidence interval included the null value and the difference was not statistically significant; P-CAB had the highest numerical P-score ranking; combination regimens (PPI + MP, P-CAB + MP) also yielded numerically better healing outcomes. At 8 weeks, PPI + MP had the highest numerical P-score ranking, though no regimen achieved statistically superior healing compared with PPI. For delayed bleeding and perforation, all regimens demonstrated comparable safety profiles, while H2RA-based treatments showed a non-significant trend toward higher complication risk. Most pairwise comparisons were supported by low-to-moderate certainty evidence.

**Conclusions:**

While P-CAB-based regimens and MP combinations showed numerically favorable trends for early post-ESD ulcer healing, all comparative findings are limited by mostly low-to-moderate GRADE evidence certainty, and no regimen exhibited statistically significant superiority for ulcer healing. No regimen significantly reduces delayed bleeding or perforation risk.

## 1. Introduction

Endoscopic submucosal dissection (ESD) has revolutionized the management of early-stage gastrointestinal neoplasms, offering high en-bloc resection rates and organ preservation [1–3]. gastric ESD creates larger and deeper mucosal defects, termed “artificial ulcers,” which require a prolonged period to heal. Also, these iatrogenic lesions carry a significant risk of delayed complications, for example, the post-ESD bleeding rate ranges from 4% to 12% [4].

The healing of post-ESD ulcers is highly affected by Helicobacter pylori infection, ulcer size, lesion location, etc [5]. While Proton Pump Inhibitors (PPIs) have long been the mainstay of therapy, the emergence of Potassium-Competitive Acid Blockers (P-CABs) and the adjunctive use of mucosal protective(MP) agents (e.g., rebamipide) have expanded the therapeutic landscape [6,7]. A recently published network meta-analysis has provided valuable evidence on MP combinations for post-ESD ulcers [8], but it focused exclusively on PPI-based regimens—with or without concomitant mucosal protectants— and did not evaluate P-CAB or H2RA monotherapy, Therefore, current clinical practice lacks consistent recommendations regarding the comparative efficacy of P-CABs, PPIs, H2-receptor antagonists(H2RA), or their combinations in promoting healing of post-ESD ulcers, and there is no consensus on whether combination therapy should be routinely recommended.

Given the distinct pathophysiology of gastric ESD ulcers, a focused synthesis of evidence is required. This network meta-analysis(NMA) aims to evaluate the comparative effectiveness of different pharmacological strategies to optimize post-ESD recovery.

## 2. Materials and methods

### 2.1 Study design and registration

This study is a systematic review and network meta-analysis (NMA) of randomized controlled trials (RCTs) evaluating pharmacological interventions for management of artificial ulcers and prevention of delayed bleeding after gastric endoscopic submucosal dissection (ESD). The review protocol was prospectively registered in the PROSPERO database (registration number: CRD420251057886) and was conducted following the Preferred Reporting Items for Systematic Reviews and Meta-Analyses (PRISMA) extension for network meta-analysis [9].

#### 2.1.1 Eligibility criteria.

2.1.1.1 Types of studies: We included RCTs comparing any pharmacological intervention(s) intended to promote ulcer healing or prevent delayed bleeding and perforation after ESD. Non-randomized studies, observational studies, case reports, reviews, conference abstracts without complete raw data, and animal studies were excluded.

2.1.1.2 Participants: Adult patients (≥18 years) who underwent gastric ESD were eligible for inclusion.

2.1.1.3 Interventions and comparators: Any pharmacological regimen intended to reduce gastric acidity, protect gastrointestinal mucosa, or improve post-ESD clinical outcomes were eligible, including but not limited to PPIs(oral or intravenous), P-CABs, H2RAs, MPs, and combined regimens (e.g., PPIs combined with MPs).

2.1.1.4 Outcomes: Primary outcomes included two indicators: (1) delayed bleeding or perforation after gastric ESD. Delayed bleeding was defined as hematemesis requiring endoscopic hemostasis, blood transfusion or hospital readmission; perforation was confirmed by abdominal computed tomography (CT) or clinical manifestations of peritonitis. (2) Post-ESD ulcer healing, which was assessed according to the Sakita-Miwa endoscopic staging system, and the proportion of patients achieving S1 and S2 File stage was extracted as the main indicator. For trials reporting multiple follow-up time points, we extracted data at 4 weeks and 8 weeks after ESD preferentially.

#### 2.1.2 Literature search strategy.

We systematically searched PubMed/MEDLINE, Embase, and the Cochrane Central Register of Controlled Trials (CENTRAL) from database inception to December 10, 2025. The search strategy combined Medical Subject Headings (MeSH) and free-text terms related to gastric ESD and acid-suppressive/mucosal protective drugs. The detailed search formula was as follows:

(“endoscopic submucosal dissection”[MeSH Terms] OR “endoscopic submucosal dissection” OR “ESD”) AND(“proton pump inhibitor*” OR “PPI” OR “potassium-competitive acid blocker*” OR “vonoprazan” OR “P-CAB” OR “H2-receptor antagonist*” OR “H2RA” OR “rebamipide” OR “sucralfate” OR “mucosal protect*” OR “acid suppress*” OR “acid blockade”) AND (“randomized” OR “randomised” OR “randomized controlled trial” OR “random allocation” OR “randomly”)

#### 2.1.3 Study selection.

All retrieved literature records were imported into reference management software to remove duplicates, and non-English publications were excluded. Two independent reviewers initially screened titles and abstracts to identify potentially eligible studies. Full texts of candidate articles were further retrieved and assessed against the predefined inclusion and exclusion criteria. Any disagreements were resolved through group discussion; if consensus could not be reached, a third senior reviewer made the final judgment. A PRISMA flow diagram was used to summarize the number of retrieved, screened, excluded and included studies (Fig 1).

**Fig 1:**
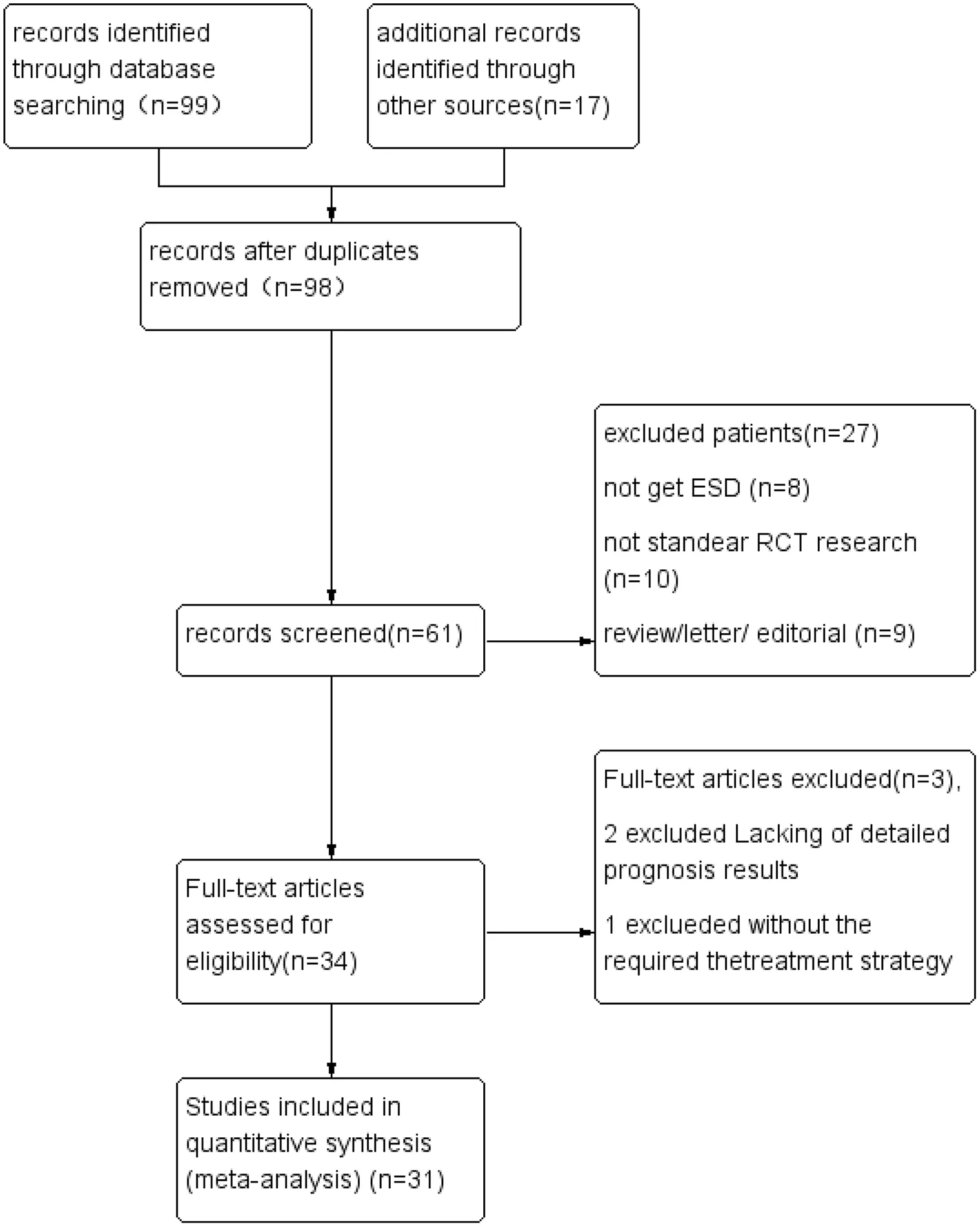
PRISMA flow diagram of study selection process.

#### 2.1.4 Data extraction.

A standardized data extraction form was designed and pre-tested before formal extraction. Two reviewers independently extracted data from included studies, and inconsistencies were resolved by discussion or adjudication by a third reviewer. The extracted information contained basic study characteristics (first author, publication year, study country), study design, sample size of each intervention arm, follow-up duration, outcome assessment time points, event numbers and total cases for binary outcomes, as well as mean values and standard deviations for continuous outcomes (if reported).

#### 2.1.5 Risk of bias assessment.

The Cochrane Risk of Bias (RoB) tool (assessed via Review Manager 5.4 software) was used to evaluate the methodological quality and risk of bias of all included RCTs. Seven bias domains were assessed: (1) random sequence generation (selection bias), (2) allocation concealment (selection bias), (3) blinding of participants and personnel (performance bias), (4) blinding of outcome assessment (detection bias), (5) incomplete outcome data (attrition bias), (6) selective outcome reporting (reporting bias), and (7) other potential biases.

Each domain was rated as low risk, unclear risk, or high risk of bias. An overall risk of bias judgment was assigned for each primary outcome. Discrepancies during assessment were resolved by discussion or consultation with a third reviewer. The detailed results of risk of bias assessment were presented in tables and figures (Fig 2).

**Fig 2:**
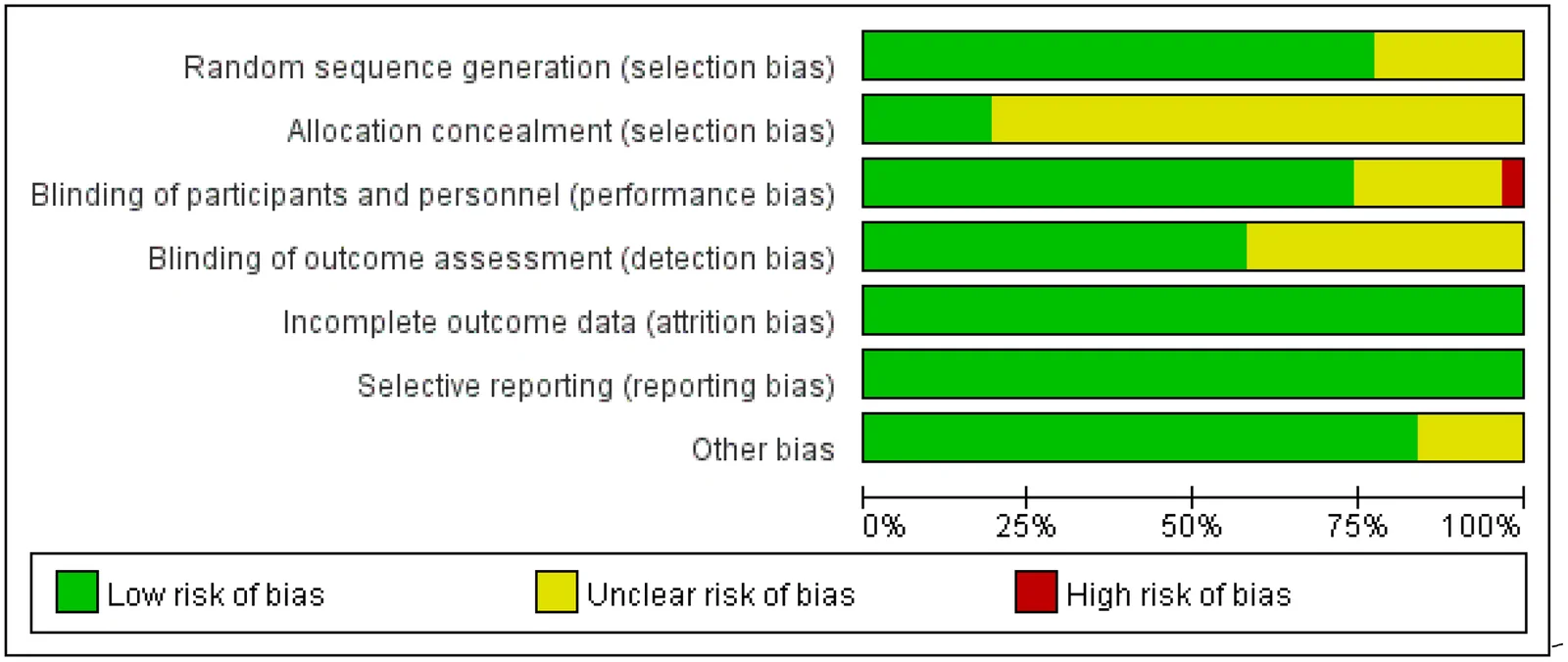
Risk of bias graph showing each risk of bias item as percentages across all included studies.

#### 2.1.6 Assessment of transitivity.

The transitivity assumption is a key premise for valid network meta-analysis. We evaluated the distribution of potential effect modifiers across different comparison groups, including ulcer size, *Helicobacter pylori* infection status, gastric lesion location, and use of antithrombotic drugs. Since baseline clinical variables were incompletely reported in partial studies, formal quantitative comparison was not performed. No obvious violation of the transitivity assumption was identified from the available baseline data and unified eligibility criteria. However, robust quantitative assessment of transitivity was not possible due to incomplete reporting of key effect modifiers, and residual imbalance in unreported variables may affect the validity of indirect comparisons.

#### 2.1.7 Data synthesis and statistical analysis.

All statistical analyses were performed using R software (version 4.3.0) based on a frequentist framework of network meta-analysis, using the “netmeta” package (version 2.1−0).

For binary outcomes (ulcer healing, delayed bleeding and perforation), event counts and total sample sizes of each arm were extracted. Odds ratios (OR) with 95% confidence intervals (CIs) were calculated for all binary outcomes including ulcer healing, delayed bleeding, and perforation; OR was chosen for safety outcomes given the low event rates.;

For continuous outcomes, mean differences (MD) with 95% CIs were calculated. If only median and interquartile range were reported, validated methods were used to estimate corresponding mean values and standard deviations.

Network geometry plots were constructed to visualize direct comparative relationships among different interventions. Considering substantial between-study heterogeneity, the random-effects model was adopted as the primary analytical model, as prespecified in the registered protocol based on expected between-study heterogeneity; the fixed-effects model was additionally applied for main effect pooling, subgroup analysis and sensitivity analysis to verify the robustness of results.

Global inconsistency was evaluated using the design-by-treatment interaction model, and node-splitting analysis was used to assess local inconsistency between direct and indirect evidence. P value < 0.05 was defined as statistically significant inconsistency.

Heterogeneity across pairwise comparisons was quantified by τ^2^ and I^2^ statistics.

P-scores (frequentist equivalent to SUCRA) were calculated to rank the efficacy and safety of different interventions for each outcome; a higher P-score represented a better therapeutic effect. P-scores were calculated using the netrank() function in the netmeta package (method = “P-score”). SUCRA values were additionally derived using the same netrank() function with method = “SUCRA,” based on 1000 resamples with a fixed seed for reproducibility. Both metrics are generated from the same frequentist statistical framework and produced highly consistent ranking orders; we report both for transparency and comparability with previous network meta-analyses.

Leave-one-out sensitivity analysis was performed by sequentially excluding each single study from the network to test the stability of pooled results.

Comparison-adjusted funnel plots were planned to assess publication bias and small-study effects when the number of included studies was sufficient. The GRADE (Grading of Recommendations, Assessment, Development and Evaluations) system was used to rate the certainty of evidence for all primary outcomes. Evidence quality was classified into four grades (high, moderate, low, and very low) according to five domains: risk of bias, inconsistency, indirectness, imprecision, and publication bias.

## 3. Result

### 3.1 Study inclusion, network structures and assessment of transitivity

A total of 31 randomized controlled trials (RCTs) [10–40] (Table 1) were included after removal of duplicates and screening of titles, abstracts, and full texts.

**Table 1:** Characteristics of included randomized controlled trials.

	StudyID	Region	cases	Treatment regimens	Primary outcomes	Assessment method	Follow-up duration
1	Yan2019	China	133/137	PPI VS PPI + MP	4-week ulcer reduction rate	Endoscopic examination	4 weeks, 8 weeks
2	Komor2019	Japan	20/20	PPI VS P-CAB	4-week ulcer reduction rate	Endoscopic examination	4 weeks
3	Ohya2010	Japan	31/29	PPI VS H_2_RA	4-week ulcer size reduction rate	Endoscopic examination	4 weeks
4	Kawai2021	Japan	83/85	PPI VS P-CAB	4-week artificial ulcer healing rate	Endoscopic examination	4 weeks, 8 weeks
5	Fujiwara2011	Japan	31/30	PPI VS PPI + MP	8-week (56-day) ulcer healing rate	Endoscopic examination	8 weeks (56 days)
6	Shin2011	Korea	139/126	PPI VS P-CAB	4-week ulcer healing rate	Endoscopic examination	4 weeks, 8 weeks (for unhealed ulcers)
7	Kato2010	Japan	31/31	PPI VS PPI + MP	4-week (28-day) ulcer healing rate	Endoscopic examination	4 weeks (28 days)
8	Araki2012	Japan	42/45	PPI VS PPI + MP	4-week (28-day) artificial ulcer healing rate	Endoscopic examination	4 weeks (28 days)
9	Hira2018	Japan	68/61	PPI VS P-CAB	4-week and 8-week ulcer area and shrinkage ratio	Endoscopic examination	4 weeks, 8 weeks
10	Jung2021	Korea	79/100	PPI + MP	4-week ulcer healing rate	Endoscopic examination	4 weeks
11	Ko2019	Korea	94/98	PPI	Ulcer contraction rate, scar-change rate at 4 weeks	Endoscopic examination	4 weeks
12	Uedo2007	Japan	73/70	PPI VS H_2_RA	Incidence of delayed bleeding from ESD-induced ulcer	Clinical follow-up (hematemesis/melena monitoring, hemoglobin test), endoscopic hemostasis confirmation	8 weeks
13	Bang2018	Korea	77/78	PPI	Ulcer healing rate at 4 and 8 weeks	Endoscopic examination	8 weeks (follow-up at 4 weeks and 8 weeks)
14	Imada2011	Japan	62/61	PPI VS H_2_RA	Incidence of delayed bleeding, ulcer healing rate at 8 weeks	Clinical follow-up (bleeding monitoring, hemoglobin test), endoscopic examination to evaluate ulcer healing	8 weeks
15	Tsuchiya2017	Japan	41/39	PPI VS P-CAB	8-week ulcer cure rate (scar stage rate)	Endoscopic examination	8 weeks (follow-up at 1, 2, 4, 6, 8 weeks)
16	Ishii2018	Japan	26/27	PPI + MP VS P-CAB + MP	Ulcer shrinkage rate at 4 and 8 weeks	Endoscopic examination	8 weeks (follow-up at 4 weeks and 8 weeks)
17	Takayama2013	Japan	44/45	PPI VS MP	Ulcer healing rate (regression to S-stage) at 4 and 8 weeks	Endoscopic examination	8 weeks (follow-up at 4 weeks and 8 weeks)
18	Hamada2019	Japan	70/69	PPI VS P-CAB	8 weeks Incidence of delayed bleeding from ESD-induced gastric ulcer	Clinical follow-up, endoscopic confirmation of active bleeding	8 weeks
19	Tomita2012	Japan	77/79	PPI VS H_2_RA	8 weeks Incidence of delayed bleeding from ESD-induced ulcer	Clinical follow-up, endoscopic hemostasis confirmation	8 weeks
20	Kawano2011	Japan	42/43	PPI	Ulcer healing rate, ulcer reduction ratio, prevention of bleeding, quality of life	Endoscopic examination,	4 weeks (28 days) and 8 weeks (56 days) post-ESD
21	Ichida2019	Japan	39/43	PPI + MP VS P-CAB + MP	Ulcer scar rate, gastric ulcer reduction rate at 4 and 8 weeks post-ESD	Endoscopic examination	4 weeks and 8 weeks post-ESD
22	LEE2020	Korea	52/52/52	PPI VS PPI + MP VS H_2_RA + MP	Ulcer residual ratio, ulcer stage (S stage rate) at 28 days post-ESD	Endoscopic examination	28 days (4 weeks) post-ESD
23	Ahn2015	Korea	40/39	PPI VS PPI + MP	Proportion of ulcer size reduction at 4 weeks post-ESD	Endoscopic examination	4 weeks post-ESD
24	Kato2023	Japan	121	P-CAB	Proportion of complete closure of post-ESD mucosal wound at 24 weeks post-ESD	Esophagogastroduodenoscopy	8 weeks and 24 weeks post-ESD
25	Aral2014	Japan	33	PPI + MP	Ulcer healing rate at 4 weeks post-ESD	Endoscopic examination	4 weeks post-ESD
26	Takahashi2016	Japan	112/121	PPI VS P-CAB	Shrinking rate of ESD-induced ulcers on day 8 and day 28 post-ESD	Endoscopic examination	8 days and 28 days (4 weeks) post-ESD
27	Kim2024	Korea	68/76	PPI VS P-CAB	Healing rates of ESD-induced artificial ulcers at 4 and 8 weeks post-ESD	Gastroscopic examination	4 weeks and 8 weeks post-ESD
28	Shibata2025	Japan	60/59	PPI VS P-CAB	Delayed bleeding rate within 8 weeks post-ESD	Endoscopic examination	8 weeks post-ESD
29	Yang2025	China	68/64	P-CAB VS P-CAB + MP	Ulcer healing rate (4/8 weeks), scar quality	Endoscopic examination	4 weeks, 8 weeks
30	Kobayashi2012	Japan	85/85	PPI VS PPI + MP	Ulcer healing rate (marginal + basal), factors associated with delayed healing	Endoscopic examination	4-6 weeks
31	Nakamura2016	Japan	25/25	PPI VS PPI + MP	Ulcer scar stage (S stage) achievement rate (4/8 weeks)	Endoscopic examination	4 weeks, 8 weeks

Seven treatment strategies were evaluated, including proton pump inhibitors (PPI), potassium-competitive acid blockers (P-CAB), histamine-2 receptor antagonists (H2RA), mucosal protective agents (MP), and combination regimens incorporating mucosal protection. The evidence network was well connected without isolated nodes, with PPI representing the dominant comparator across the majority of direct comparisons (Fig 3).

**Fig 3:**
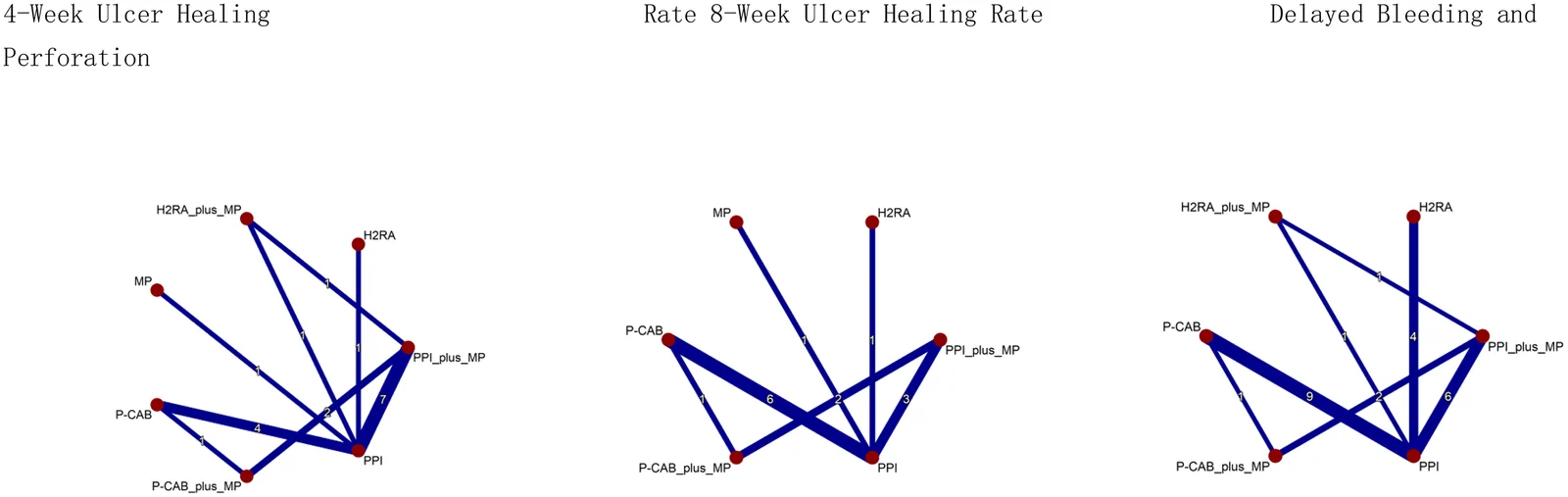
Network geometry of comparisons between 7 regimens for ulcer healing after ESD.

Potential effect modifiers considered relevant to ulcer healing after gastric ESD were evaluated across the included studies.

Ulcer size was the most consistently reported variable. Among studies providing baseline measurements, mean ulcer diameter generally ranged from approximately

30 to 40 mm, whereas ulcer area typically ranged from 700 to 1,500 mm^2^. No systematic differences in ulcer size were observed between treatment comparisons.

Helicobacter pylori infection status was reported in a subset of studies. Although reporting methods varied, most studies indicated comparable infection rates between treatment groups or reported no statistically significant between-group differences.

Lesion location was reported in several studies and was generally balanced between treatment arms, with no consistent differences in the distribution of upper, middle, or lower gastric lesions.

The use of antithrombotic agents was either an exclusion criterion or infrequently reported. In studies reporting antithrombotic exposure, the proportion of users was low and did not differ substantially between treatment groups.

Overall, no obvious violation of the transitivity assumption was identified from the available baseline data. However, robust quantitative assessment of transitivity was not possible due to incomplete reporting of key effect modifiers (H. pylori status, ulcer size, lesion location, antithrombotic drug use), and residual imbalance in unreported variables may affect the validity of indirect comparisons.

### 3.2 Four-week ulcer healing rate

#### 3.2.1 Comparative efficacy.

This network meta-analysis included 16 studies comparing 7 treatment strategies across 18 pairwise comparisons. In the random-effects model, no intervention demonstrated statistically significant superiority over PPI monotherapy for 4-week ulcer healing. The point estimate favored P-CAB compared with PPI (OR 1.61, 95% CI 0.96–2.68; P = 0.069), but the confidence interval included 1.0 and the difference was not statistically significant.

PPI combined with mucosal protective agents also showed numerically improved healing efficacy relative to PPI alone (OR 1.24, 95% CI 0.81–1.92), although the difference did not reach statistical significance. Similarly, P-CAB + MP demonstrated a favorable treatment estimate (OR 1.34, 95% CI 0.53–3.39). By contrast, MP alone, H2RA, and H2RA+MP failed to demonstrate superiority over conventional PPI therapy (Table 2).

**Table 2:** Meta-analysis results of ulcer healing and postoperative complications.

	Treatment	Odds Ratio (OR)	95% Confidence Interval (CI)	P-value
	PPI (reference)	–	–	–
4-week Ulcer Healing Rate	P-CAB	1.61	0.96 to 2.68	0.069
	PPI + MP	1.24	0.81 to 1.92	0.323
	P-CAB + MP	1.34	0.53 to 3.39	0.535
	MP	1.33	0.40 to 4.42	0.638
	H2RA	1.08	0.17 to 6.92	0.938
	H2RA + MP	0.66	0.23 to 1.88	0.438
8-week Ulcer Healing Rate	MP	1.4	0.27 to 7.30	0.6897
	PPI + MP	1.29	0.71 to 2.37	0.4052
	P-CAB + MP	1.2	0.49 to 2.96	0.6901
	H2RA	0.98	0.21 to 4.56	0.9823
	P-CAB	0.87	0.58 to 1.30	0.4917
Delayed bleeding/perforation	P-CAB + MP	0.866	0.094 to7.986	0.8991
	PPI + MP	0.891	0.273 to 2.910	0.8481
	P-CAB	0.997	0.382 to 2.605	0.9955
	H2RA+MP	2.712	0.210 to 34.994	0.4444
	H2RA	3.769	0.936 to 15.181	0.062

As a sensitivity analysis, the common-effects model yielded a statistically significant estimate for P-CAB versus PPI (OR 1.64, 95% CI 1.15–2.32; P = 0.006). This difference from the primary random-effects analysis likely reflects differential study weighting (common-effect models give greater weight to larger studies), and this result should not be interpreted as independent evidence of treatment superiority.

Pairwise treatment comparisons are summarized in the league table (Table 3)

**Table 3:** Pairwise treatment comparisons (league table).

4-week Ulcer Healing Rate									
Treatment	PPI		P-CAB	PPI+MP	P-CAB +MP	MP		H2RA	H2RA +MP
**PPI**	—								
**P-CAB**	1.61 (0.96 to 2.68)		—						
**PPI +MP**	1.24 (0.81 to 1.92)		0.77 (0.43 to 1.38)	—					
**P-CAB +MP**	1.34 (0.53 to 3.39)		0.83 (0.31 to 2.24)	1.08 (0.41 to 2.84)	—				
**MP**	1.33 (0.40 to 4.42)		0.83 (0.23 to 2.95)	1.07 (0.31 to 3.72)	0.99 (0.26 to 3.76)	—			
**H2RA**	1.08 (0.17 to 6.92)		0.67 (0.10 to 4.43)	0.87 (0.13 to 5.71)	0.81 (0.12 to 5.40)	0.81 (0.11 to 5.93)		—	
**H2RA +MP**	0.66 (0.23 to 1.88)		0.41 (0.14 to 1.21)	0.53 (0.18 to 1.56)	0.49 (0.16 to 1.49)	0.49 (0.15 to 1.60)		0.61 (0.16 to 2.31)	—
8-week Ulcer Healing Rate									
**Treatment**		**H2RA**	**MP**	**P-CAB**	**P-CAB+MP**		**PPI**	**PPI+MP**	
**H2RA**		.	.	.	.		.	.	
**MP**		0.70 (0.07 to 6.69)	.	.	.		.	.	
**P-CAB**		1.13 (0.23 to 5.54)	1.62 (0.29 to 8.85)	.	.		.	.	
**P-CAB+MP**		0.82 (0.14 to 4.85)	1.17 (0.18 to 7.65)	0.72 (0.30 to 1.75)	.		.	.	
**PPI**		0.98 (0.21 to 4.56)	1.40 (0.27 to 7.30)	0.87 (0.58 to 1.30)	1.20 (0.49 to 2.96)		.	.	
**PPI+MP**		0.76 (0.15 to 3.95)	1.08 (0.19 to 6.29)	0.67 (0.33 to 1.35)	0.93 (0.38 to 2.28)		0.77 (0.42 to 1.42)	.	
Delayed bleeding/perforation									
		**H2RA**	**H2RA+MP**	**P-CAB**	**P-CAB+MP**		**PPI**	**PPI+MP**	
H2RA		.							
H2RA+MP		1.18 (0.20 to 6.97)	.						
P-CAB		2.23 (0.87 to 5.72)	1.89 (0.34 to 10.54)	.					
P-CAB+MP		3.62 (0.57 to 22.92)	3.07 (0.34 to 27.71)	1.62 (0.28 to 9.28)	.				
PPI		2.40 (1.16 to 4.99)	2.04 (0.40 to 10.28)	1.08 (0.59 to 1.96)	0.66 (0.12 to 3.62)		.		
PPI+MP		3.06 (1.10 to 8.53)	2.60 (0.57 to 11.76)	1.38 (0.55 to 3.46)	0.85 (0.17 to 4.26)		1.27 (0.62 to 2.62)	.	

#### 3.2.2 Ranking analysis.

P-score analysis demonstrated that P-CAB had the highest probability of being the most effective treatment for 4-week ulcer healing, followed by P-CAB + MP and PPI + MP. In contrast, H2RA-containing regimens consistently ranked among the least effective interventions (Table 4).

**Table 4:** Ranking of treatment efficacy based on P-score and SUCRA values.

	Rank	Treatment	P-score	SUCRA (%)
4-week Ulcer Healing Rate	1	P-CAB	0.86	86.20
	2	P-CAB + MP	0.72	71.50
	3	MP	0.71	70.80
	4	PPI + MP	0.62	62.40
	5	H2RA	0.51	51.10
	6	PPI (Reference)	0.50	49.50
	7	H2RA + MP	0.09	8.50
8-week Ulcer Healing Rate	1	PPI + MP	0.66	66.44
	2	MP	0.62	61.69
	3	P-CAB + MP	0.58	57.61
	4	H2RA	0.44	44.33
	5	PPI	0.43	43.11
	6	P-CAB	0.27	26.81
Delayed bleeding/perforation	1	PPI + MP	0.68	67.58
	2	P-CAB + MP	0.65	64.67
	3	PPI	0.62	62.36
	4	P-CAB	0.62	61.96
	5	H2RA+MP	0.29	29.38
	6	H2RA	0.14	14.04

Differences between the leading regimens were relatively small. Therefore, treatment rankings should be interpreted cautiously.

#### 3.2.3 Heterogeneity and inconsistency.

Moderate heterogeneity was observed across studies (τ^2^ = 0.159; I^2^ = 45.5%). Nevertheless, no statistically significant inconsistency between direct and indirect evidence was detected using the design-by-treatment interaction model (Q = 0.05, P = 0.976).

Node-splitting analyses demonstrated no statistically significant disagreement between direct and indirect evidence for any closed loop (all P > 0.05), indicating acceptable local consistency across the network (Fig 4a).

**Fig 4:**
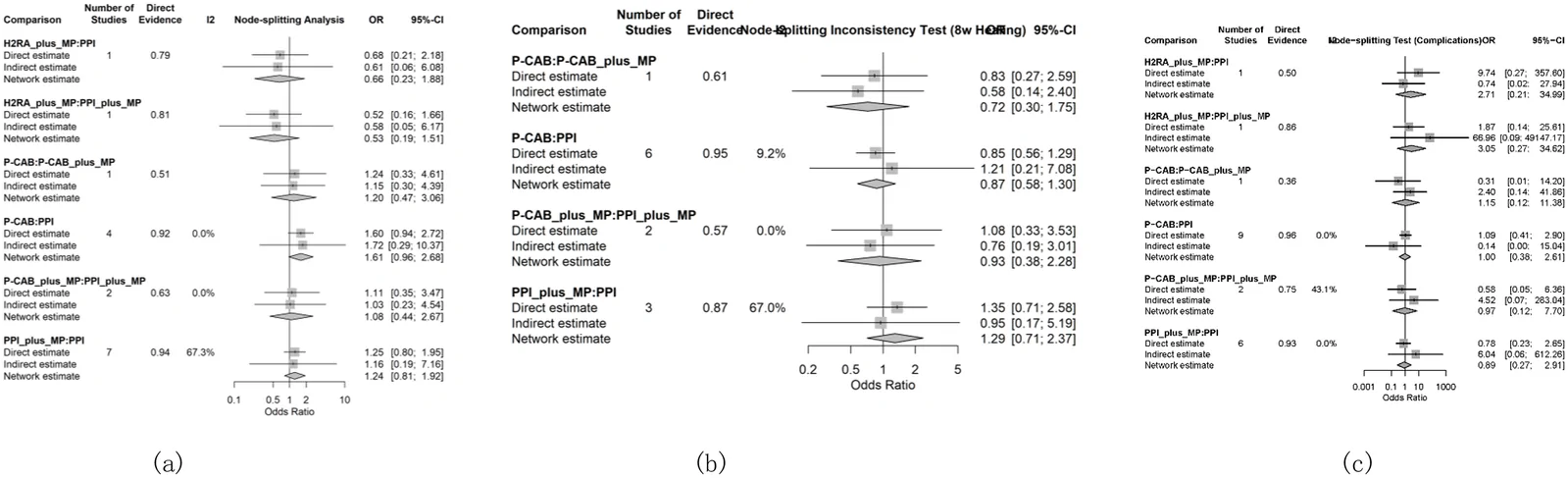
Node-splitting forest plots for inconsistency assessment 4/8-week ulcer healing rates and delayed bleeding /perforation after ESD.

Contribution matrix analysis demonstrated that the majority of network estimates were driven by direct evidence from comparisons involving PPI monotherapy. The largest contributions originated from the PPI versus PPI + MP and PPI versus P-CAB comparisons, indicating that these comparisons served as the principal sources of information within the network.(Fig 5a)

**Fig 5:**
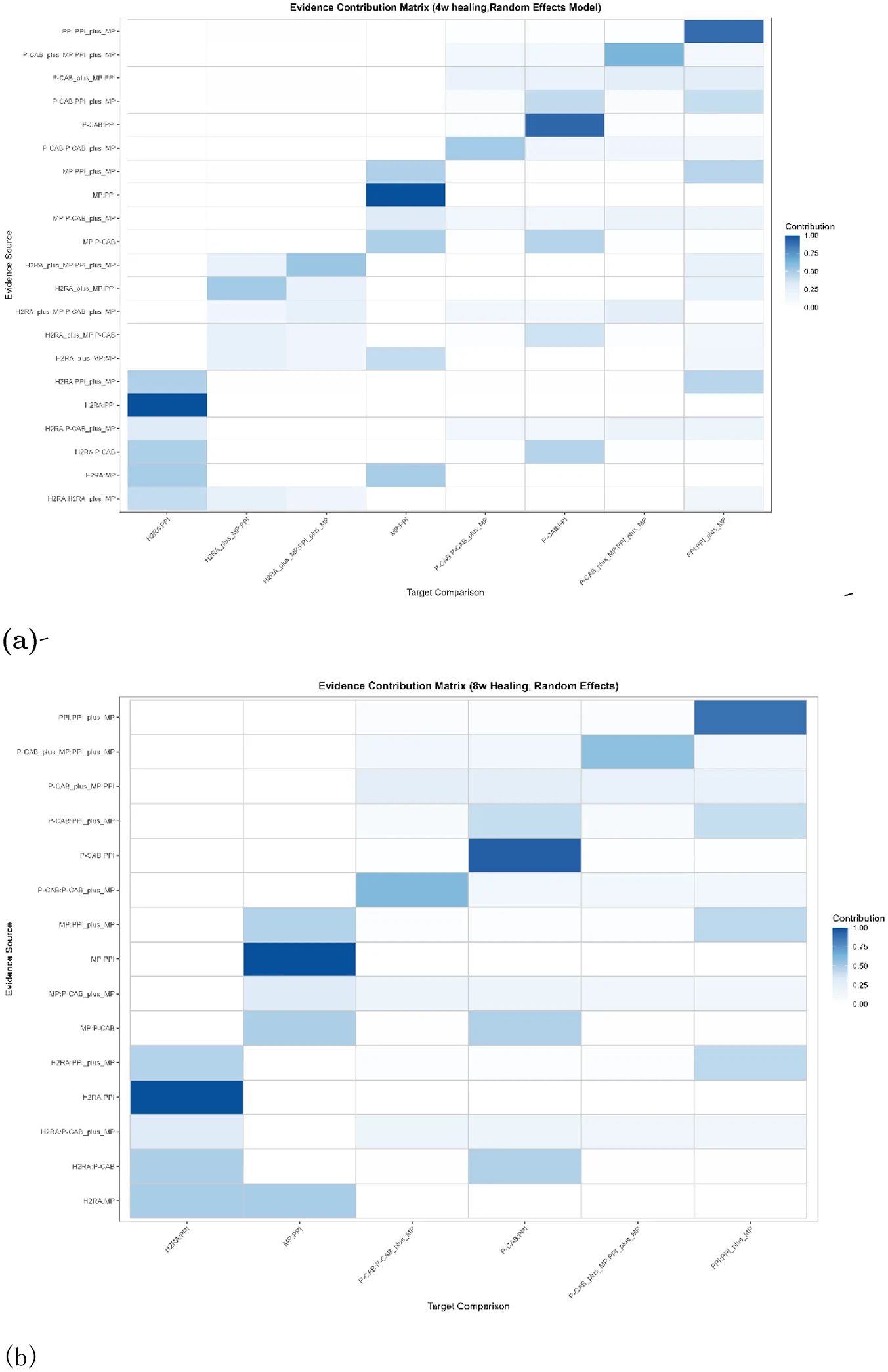
Contribution matrices for direct and indirect evidence across the network. (a) 4-week ulcer healing outcome. (b) 8-week ulcer healing outcome. The contribution matrix quantifies the proportion of direct versus indirect evidence contributing to each pairwise comparison in the network meta-analysis.

#### 3.2.4 Sensitivity analysis.

Sequential leave-one-out sensitivity analyses demonstrated stable pooled estimates for 4-week ulcer healing. Exclusion of individual studies did not materially alter either the direction or magnitude of the pooled treatment effects.

Furthermore, the superiority trend favoring P-CAB remained consistent throughout sensitivity analyses, indicating that the primary findings were not disproportionately influenced by any single study.

#### 3.2.5 Publication bias.

Because no treatment comparison included ≥10 studies, assessment of small-study effects and publication bias was considered inappropriate and therefore was not conducted.

### 3.3 Eight-week ulcer healing rate

#### 3.3.1 Comparative efficacy.

At 8 weeks, this network meta‑analysis included 14 two‑arm studies comprising 6 treatment strategies (PPI, P‑CAB, PPI + MP, P‑CAB + MP, MP, and H2RA), yielding 14 pairwise comparisons across 6 distinct designs, differences among treatment strategies became less pronounced. In the random-effects model, none of the evaluated interventions demonstrated statistically significant superiority over PPI monotherapy. Compared with PPI, P-CAB yielded an OR of 0.87 (95% CI 0.58–1.30; P = 0.492), whereas PPI + MP demonstrated an OR of 1.29 (95% CI 0.71–2.37; P = 0.405).(Table 2)

Likewise, neither P-CAB + MP nor MP monotherapy significantly improved late-stage ulcer healing. These findings suggest that the potential early healing advantage associated with intensified acid suppression may diminish over time as mucosal regeneration progresses naturally.

Pairwise treatment comparisons are summarized in the league table (Table 3)

#### 3.3.2 Ranking analysis.

Despite the absence of statistically significant pairwise differences, ranking analysis indicated that PPI + MP had the highest probability of being the optimal treatment for 8-week healing, followed by MP alone and P-CAB + MP (Table 4).

By contrast, P-CAB monotherapy demonstrated the lowest ranking probability. Given the relatively wide confidence intervals and limited direct evidence, these ranking results should be interpreted cautiously.

#### 3.3.3 Heterogeneity and inconsistency.

The 8-week analysis demonstrated relatively low heterogeneity (τ^2^ = 0.078; I^2^ = 24.9%). No statistically significant inconsistency was observed in either the design-by-treatment interaction model or node-splitting analyses (all P > 0.05), indicating good agreement between direct and indirect evidence.

Node-splitting analyses demonstrated no statistically significant disagreement between direct and indirect evidence for any closed loop (all P > 0.05), indicating acceptable local consistency across the network (Fig 4b).

Contribution matrix analysis demonstrated that the majority of network estimates were driven by direct evidence from comparisons involving PPI monotherapy. The largest contributions originated from the PPI versus PPI + MP and PPI versus P-CAB comparisons, indicating that these comparisons served as the principal sources of information within the network. (Fig 5b).

#### 3.3.4 Sensitivity analysis.

Leave-one-out sensitivity analyses demonstrated stable pooled estimates for 8-week healing outcomes. Sequential exclusion of individual studies did not substantially affect treatment rankings or overall effect estimates.

In subgroup analyses restricted to larger studies, heterogeneity was markedly reduced, suggesting that smaller studies may have contributed disproportionately to inter-study variability.

#### 3.3.5 Publication bias.

Formal evaluation of publication bias for the 8-week ulcer healing outcome was not conducted because most treatment comparisons included fewer than 10 studies, making funnel plot asymmetry tests unreliable.

### 3.4 Postoperative complications: Delayed bleeding and perforation

#### 3.4.1 Comparative safety.

Across all treatment groups, the incidence of major post-ESD complications (delayed bleeding and perforation) was low. The analysis pooled 22 studies covering 6 treatment strategies (PPI, P‑CAB, PPI + MP, P‑CAB + MP, H2RA, and H2RA + MP) and contributed 24 pairwise comparisons across 6 distinct designs.Comparative analysis demonstrated no statistically significant increase in complication risk associated with P-CAB-containing regimens compared with conventional PPI therapy.

Similarly, combination therapy with mucosal protective agents did not significantly reduce adverse event rates relative to acid-suppressive monotherapy. Although H2RA-containing regimens tended to demonstrate less favorable safety estimates, the corresponding confidence intervals were wide because of the limited number of reported events.(Table 2)

Pairwise treatment comparisons are summarized in the league table (Table 3).

#### 3.4.2 Ranking analysis.

Ranking analysis suggested that combination regimens incorporating mucosal protective agents were associated with the most favorable safety profiles, whereas H2RA-containing therapies ranked lowest for complication prevention. Nevertheless, the low overall event rate limited the certainty of these rankings. (Table 4).

#### 3.4.3 Heterogeneity and inconsistency.

Moderate heterogeneity was observed in the complication analysis. No statistically significant inconsistency between direct and indirect evidence was detected within the safety network.

Node-splitting analysis presented in the forest plot indicated that direct and indirect evidence were consistent for all treatment comparisons (P > 0.05). (Fig 4c).

The network for gastric ESD postoperative complications had a simple structure. Two major comparisons relied on over 90% direct evidence, while direct evidence ranged from 0.36 to 0.86 for the rest; an evidence contribution matrix was not available due to insufficient triangular loops. Node-splitting tests found no significant inconsistency between direct and indirect evidence across all comparisons (P > 0.05), confirming good consistency of this network meta-analysis.

#### 3.4.4 Sensitivity analysis.

Sensitivity analyses demonstrated stable complication estimates after sequential exclusion of individual studies. Notably, exclusion of the study by Tomita et al. substantially reduced heterogeneity, suggesting that this study represented a major contributor to inter-study variability.

#### 3.4.5 Publication bias.

Because of the relatively small number of complication events and the presence of multiple zero-event studies, formal assessment of publication bias for safety outcomes was considered unreliable and therefore was not formally performed.

### 3.5 Ulcer reduction rate

Because most included studies did not report variance measures for ulcer reduction rates, quantitative pooling using conventional meta-analytic methods was not feasible. Therefore, a descriptive synthesis was performed.

Across studies reporting ulcer size reduction,numerical reductions in ulcer area at 4 weeks were generally larger with P-CAB + MP in the studies that reported this outcome, with several studies reporting reduction rates exceeding 90%. PPI + MP and P-CAB monotherapy also showed numerically higher reduction rates compared with conventional PPI therapy in the available studies.

By contrast, PPI monotherapy generally achieved reduction rates ranging from approximately 80% to 90%. These are descriptive numerical observations only and should not be interpreted as evidence of comparative treatment superiority, given the absence of quantitative pooling and potential differences in patient characteristics and measurement methods across studies.

### 3.6 Certainty of evidence

Publication bias judgments in the GRADE assessment were based on qualitative considerations, including study sample size distribution, direction of effect estimates, and potential small-study effects, rather than formal statistical testing, which was not feasible due to the limited number of studies per comparison. According to the GRADE framework, the certainty of evidence for the comparison between P-CAB and PPI was rated as moderate. Evidence certainty for combination regimens and H2RA-based therapies ranged from low to very low owing to imprecision, limited direct evidence, and inter-study heterogeneity, Accordingly, all numerical efficacy rankings should be interpreted with caution. Comparisons rated as low or very low certainty do not support firm clinical conclusions, whereas moderate-certainty comparisons may support directional conclusions with acknowledgment that the true effect may differ from the estimated effect. (Table 5)

**Table 5:** GRADE assessment of evidence certainty.

	Comparison	Risk of bias	Inconsistency	Indirectness	Imprecision	Publication bias	Overall certainty
4-week Ulcer Healing Rate	P-CAB vs PPI	Not serious	Not serious	Not serious	Serious	Undetected	Moderate
	PPI + MP vs PPI	Not serious	Not serious	Not serious	Not serious	Undetected	Moderate
	P-CAB + MP vs PPI	Serious	Serious	Not serious	Serious	Undetected	Low
	MP vs PPI	Serious	Serious	Not serious	Very serious	Suspected	Low
	H2RA vs PPI	Serious	Very serious	Not serious	Very serious	Suspected	Low
	H2RA+MP vs PPI	Serious	Very serious	Not serious	Very serious	Suspected	Very low
8-week Ulcer Healing Rate	P-CAB vs PPI	Not serious	Not serious	Not serious	Serious	Undetected	Moderate
	PPI + MP vs PPI	Not serious	Not serious	Not serious	Not serious	Undetected	Moderate
	P-CAB + MP vs PPI	Serious	Serious	Not serious	Serious	Undetected	Low
	MP vs PPI	Serious	Serious	Not serious	Very serious	Suspected	Low
	H2RA vs PPI	Serious	Serious	Not serious	Very serious	Suspected	Low
	H2RA+MP vs PPI	Serious	Very serious	Not serious	Very serious	Suspected	Very low
Delayed bleeding / perforation	P-CAB vs PPI	Not serious	Not serious	Not serious	Serious	Undetected	Moderate
	PPI + MP vs PPI	Not serious	Not serious	Not serious	Serious	Undetected	Moderate
	P-CAB + MP vs PPI	Serious	Serious	Not serious	Serious	Suspected	Low
	H2RA vs PPI	Serious	Serious	Not serious	Very serious	Suspected	Very low

## 4. Discussion

This network meta-analysis comprehensively evaluated the comparative efficacy of seven pharmacological regimens for gastric post-ESD ulcers, focusing on mucosal healing, ulcer reduction, and delayed bleeding prevention. Our analysis revealed time-dependent trends in therapeutic effects across different regimens. Specifically, the point estimates for P-CAB monotherapy and PPI plus MP favored improved short-term (4-week) mucosal healing, although neither reached statistical significance in the primary analysis. At the 8-week follow-up, no monotherapy or combination regimen achieved statistically superior ulcer healing compared with PPI. Notably, no regimen demonstrated statistically significant superiority over PPI monotherapy for ulcer reduction or delayed bleeding/perforation prevention, although H2RA-based regimens showed a non-significant trend toward increased bleeding risk. These findings provide updated evidence to guide post-ESD pharmacological management.

The observed short-term benefit of P-CAB monotherapy (OR=1.61, 95% CI: 0.96–2.68, p = 0.069) and PPI plus MP (OR=1.24, 95% CI: 0.81–1.92) is consistent with their pharmacodynamic properties. P-CAB provides rapid and sustained acid suppression through reversible potassium-competitive inhibition of H + /K + -ATPase, whereas PPIs require acid activation for full efficacy [41]. In addition, MPs may enhance epithelial repair through promotion of prostaglandin synthesis, angiogenesis, and reduction of oxidative stress [42–44]. However, subgroup analysis suggested that the apparent benefit of PPI plus MP may be influenced by small-sample studies, as the effect was not confirmed in larger cohorts.

For 8-week mucosal healing, no regimen demonstrated statistically significant superiority over PPI. Although P-score ranking suggested that PPI plus MP ranked highest (P-score = 0.6644), followed by MP monotherapy and P-CAB plus MP, these rankings should be interpreted cautiously given the wide confidence intervals and sparse data in several comparisons. This finding is consistent with previous evidence suggesting that MP-based combinations may facilitate mucosal repair by targeting both acid suppression and epithelial regeneration pathways [42–45]

Despite improvements in early mucosal healing, no significant differences were observed in ulcer reduction at either 4 or 8 weeks. This contrasts with prior reports suggesting faster ulcer shrinkage with P-CAB-based therapy [15,27,42,46]. This discrepancy may be explained by heterogeneity in baseline ulcer size and the selected time points, which may not capture early-phase healing dynamics.

Regarding delayed bleeding and perforation, no regimen demonstrated statistically significant benefit over PPI monotherapy. H2RA-containing regimens showed a non-significant trend toward increased risk of adverse events (OR=3.77, 95% CI: 0.94–15.18, p = 0.062), although this finding should be interpreted cautiously due to limited event numbers and wide confidence intervals.

A detailed side-by-side comparison with the recently published network meta-analysis [8] is provided in Supplement Table1, positioning our work as an updated and expanded evidence synthesis for post-ESD pharmacological management. Compared with the prior analysis, our study applies stricter inclusion criteria that exclude non-human studies, covers seven mainstream clinical regimens including P-CAB and H2RA monotherapy, and extends the literature search to the end of 2025, providing a more clinically relevant and methodologically rigorous evidence base. This study has several key strengths. First, only randomized controlled trials with full-text availability were included, ensuring a homogeneous evidence base. Second, we comprehensively evaluated both acid-suppressant monotherapy and mucosal protector combination regimens, extending prior meta-analyses that focused exclusively on limited PPI-based comparisons. Third, sensitivity and subgroup analyses confirmed the robustness of the main findings. Nevertheless, limitations should be acknowledged. First, several comparisons relied on indirect evidence, and although inconsistency tests were non-significant (all p > 0.05), residual bias cannot be excluded. Second, the number of H2RA studies was limited, resulting in imprecise estimates. Third, formal publication bias assessment was not feasible due to sparse data and zero-event outcomes, leaving potential small-study effects uncertain. Notably, nearly half of all pairwise comparisons in this network meta-analysis were graded low or very low certainty via GRADE, mainly attributable to sparse sample sizes for several treatment nodes and wide confidence intervals of pooled effects. While PPI plus MP ranked highest for 8-week ulcer healing based on P-score values, the moderate-certainty evidence for this comparison prohibits drawing firm conclusions about its late-stage healing advantage over single-agent acid suppression. Fifth, transitivity was only assessed qualitatively due to incomplete reporting of key effect modifiers, and residual confounding may affect the validity of indirect comparisons. Sixth, several treatment nodes were sparsely informed, and a substantial proportion of comparisons relied primarily on indirect evidence, which further limits the precision and generalizability of our findings.

## 5. Conclusion

While P-CAB-based regimens and MP combinations showed favorable trends for early post-ESD ulcer healing, all comparative findings are limited by mostly low-to-moderate GRADE evidence certainty, and no regimen exhibited statistically significant superiority for ulcer healing. No regimen significantly reduces delayed bleeding or perforation risk.

## Supporting information

S1 File PRISMA 2020 checklist. (DOCX)

S2 File PRISMA 2020 expanded checklist. (DOCX)

S3 File Supplementary Table 1. Side-by-side comparison between the present study and the previously published network meta-analysis. (DOCX)

S4 File Original data, extracted data for the network meta-analysis. (DOCX)

## References

[1] Kume K. Endoscopic therapy for early gastric cancer: Standard techniques and recent advances in ESD. World J Gastroenterol. 2014;20(21):6425–32. doi: 10.3748/wjg.v20.i21.6425 24914364 PMC4047328

[2] Rashid MU, Alomari M, Afraz S, Erim T. EMR and ESD: Indications, techniques and results. Surg Oncol. 2022;43:101742. doi: 10.1016/j.suronc.2022.101742 35370049

[3] Draganov PV, Wang AY, Othman MO, Fukami N. AGA Institute clinical practice update: Endoscopic submucosal dissection in the United States. Clin Gastroenterol Hepatol. 2019;17(1):16-25.e11. 10.1016/j.cgh.2018.07.041 30077787

[4] Yang L, Qi J, Chen W, Guo Q, Xie R, Zhao Z, et al. Low-dose PPI to prevent bleeding after ESD: A multicenter randomized controlled study. Biomed Pharmacother. 2021;136:111251. doi: 10.1016/j.biopha.2021.111251 33450486

[5] Chen DY, Chen HD, Lv XD, Huang Z, Jiang D, Li Y, et al. Outcome and risk factors of ulcer healing after gastric endoscopic submucosal dissection: A systematic review and meta-analysis. World J Gastrointest Surg. 2024;16(11):3568–77. doi: 10.4240/wjgs.v16.i11.3568 39649187 PMC11622089

[6] Gao H, Li L, Zhang C, Tu J, Geng X, Wang J, et al. Comparison of efficacy of pharmacological therapies for gastric endoscopic submucosal dissection-induced ulcers: A systematic review and network meta-analysis. Expert Rev Gastroenterol Hepatol. 2020;14(3):207–20. doi: 10.1080/17474124.2020.1731304 32063071

[7] Kamada T, Satoh K, Itoh T, Ito M, Iwamoto J, Okimoto T, et al. Evidence-based clinical practice guidelines for peptic ulcer disease 2020. J Gastroenterol. 2021;56(4):303–22. doi: 10.1007/s00535-021-01769-0 33620586 PMC8005399

[8] Chen Z, Zhan S, Yu H. A network meta-analysis of pharmacological treatments for gastric mucosal protection in artificial ulcers following endoscopic submucosal dissection of the stomach. Pharmacology. 2026;111(2):59–73. doi: 10.1159/000550164 41442424

[9] Hutton B, Salanti G, Caldwell DM, Chaimani A, Schmid CH, Cameron C, et al. The PRISMA extension statement for reporting of systematic reviews incorporating network meta-analyses of health care interventions: Checklist and explanations. Ann Intern Med. 2015;162(11):777–84. doi: 10.7326/M14-2385 26030634

[10] Yan B, Lu Z, Ge Z, Liu S, Guo X, Tian D, et al. A multicenter, randomized, controlled trial of rebamipide plus lansoprazole for the treatment of postendoscopic submucosal dissection ulcers. Clin Transl Gastroenterol. 2019;10(1):e00008. doi: 10.14309/ctg.0000000000000008 30702488 PMC6369969

[11] Komori H, Ueyama H, Nagahara A, Akazawa Y, Takeda T, Matsumoto K, et al. A prospective randomized trial of a potassium competitive acid blocker vs proton pump inhibitors on the effect of ulcer healing after endoscopic submucosal dissection of gastric neoplasia. J Int Med Res. 2019;47(4):1441–52. doi: 10.1177/0300060519828514 30816056 PMC6460617

[12] Ohya TR, Endo H, Kawagoe K, Yanagawa T, Hanawa K, Ohata K, et al. A prospective randomized trial of lafutidine vs rabeprazole on post-ESD gastric ulcers. World J Gastrointest Endosc. 2010;2(1):36–40. doi: 10.4253/wjge.v2.i1.36 21160677 PMC2998864

[13] Kawai D, Takenaka R, Ishiguro M, Okanoue S, Gotoda T, Kono Y, et al. Vonoprazan versus lansoprazole in the treatment of artificial gastric ulcers after endoscopic submucossal dissection: A randomized, open-label trial. BMC Gastroenterol. 2021;21(1):236. doi: 10.1186/s12876-021-01822-5 34022796 PMC8141195

[14] Fujiwara S, Morita Y, Toyonaga T, Kawakami F, Itoh T, Yoshida M, et al. A randomized controlled trial of rebamipide plus rabeprazole for the healing of artificial ulcers after endoscopic submucosal dissection. J Gastroenterol. 2011;46(5):595–602. doi: 10.1007/s00535-011-0372-3 21359522

[15] Shin WG, Kim SJ, Choi MH, Kim KO, Jang HJ, Park CH, et al. Can rebamipide and proton pump inhibitor combination therapy promote the healing of endoscopic submucosal dissection-induced ulcers? A randomized, prospective, multicenter study. Gastrointest Endosc. 2012;75(4):739–47. doi: 10.1016/j.gie.2011.11.004 22281110

[16] Kato T, Araki H, Onogi F, Ibuka T, Sugiyama A, Tomita E, et al. Clinical trial: Rebamipide promotes gastric ulcer healing by proton pump inhibitor after endoscopic submucosal dissection--A randomized controlled study. J Gastroenterol. 2010;45(3):285–90. doi: 10.1007/s00535-009-0157-0 19957195

[17] Araki H, Kato T, Onogi F, Ibuka T, Sugiyama A, Nakanishi T, et al. Combination of proton pump inhibitor and rebamipide, a free radical scavenger, promotes artificial ulcer healing after endoscopic submucosal dissection with dissection size >40 mm. J Clin Biochem Nutr. 2012;51(3):185–8. doi: 10.3164/jcbn.12-14 23170045 PMC3491242

[18] Hirai A, Takeuchi T, Takahashi Y, Kawaguchi S, Ota K, Harada S, et al. Comparison of the effects of vonoprazan and lansoprazole for treating endoscopic submucosal dissection-induced artificial ulcers. Dig Dis Sci. 2018;63(4):974–81. doi: 10.1007/s10620-018-4948-0 29464587

[19] Jung DH, Park JC, Lee YC, Lee SK, Shin SK, Chung H, et al. Comparison of the efficacy of polaprezinc plus proton pump inhibitor and rebamipide plus proton pump inhibitor treatments for endoscopic submucosal dissection-induced ulcers. J Clin Gastroenterol. 2021;55(3):233–8. doi: 10.1097/MCG.0000000000001357 32341237 PMC7960146

[20] Ko J, Kim SJ, Kang DH, Choi CW, Kim HW, Park SB. Dose-related healing of artificial ulcers after endoscopic submucosal dissection using esomeprazole: A randomized controlled study. Medicine (Baltimore). 2019;98(20):e15701. doi: 10.1097/MD.0000000000015701 31096515 PMC6531125

[21] Uedo N, Takeuchi Y, Yamada T, Ishihara R, Ogiyama H, Yamamoto S, et al. Effect of a proton pump inhibitor or an H2-receptor antagonist on prevention of bleeding from ulcer after endoscopic submucosal dissection of early gastric cancer: A prospective randomized controlled trial. Am J Gastroenterol. 2007;102(8):1610–6. doi: 10.1111/j.1572-0241.2007.01197.x 17403076

[22] Bang CS, Shin WG, Seo SI, Choi MH, Jang HJ, Park SW, et al. Effect of ilaprazole on the healing of endoscopic submucosal dissection-induced gastric ulcer: Randomized-controlled, multicenter study. Surg Endosc. 2019;33(5):1376–85. doi: 10.1007/s00464-018-6412-9 30167954

[23] Imaeda H, Hosoe N, Suzuki H, Saito Y, Ida Y, Nakamura R, et al. Effect of lansoprazole versus roxatidine on prevention of bleeding and promotion of ulcer healing after endoscopic submucosal dissection for superficial gastric neoplasia. J Gastroenterol. 2011;46(11):1267–72. doi: 10.1007/s00535-011-0447-1 21805066

[24] Tsuchiya I, Kato Y, Tanida E, Masui Y, Kato S, Nakajima A, et al. Effect of vonoprazan on the treatment of artificial gastric ulcers after endoscopic submucosal dissection: Prospective randomized controlled trial. Dig Endosc. 2017;29(5):576–83. doi: 10.1111/den.12857 28267236

[25] Ishii Y, Yamada H, Sato T, Sue S, Kaneko H, Irie K, et al. Effects of vonoprazan compared with esomeprazole on the healing of artificial postendoscopic submucosal dissection ulcers: A prospective, multicenter, two-arm, randomized controlled trial. Gastroenterol Res Pract. 2018;2018:1615092. doi: 10.1155/2018/1615092 29670650 PMC5835268

[26] Takayama M, Matsui S, Kawasaki M, Asakuma Y, Sakurai T, Kashida H, et al. Efficacy of treatment with rebamipide for endoscopic submucosal dissection-induced ulcers. World J Gastroenterol. 2013;19(34):5706–12. doi: 10.3748/wjg.v19.i34.5706 24039365 PMC3769909

[27] Hamada K, Uedo N, Tonai Y, Arao M, Suzuki S, Iwatsubo T, et al. Efficacy of vonoprazan in prevention of bleeding from endoscopic submucosal dissection-induced gastric ulcers: A prospective randomized phase II study. J Gastroenterol. 2019;54(2):122–30. doi: 10.1007/s00535-018-1487-6 29943163

[28] Tomita T, Kim Y, Yamasaki T, Okugawa T, Kondo T, Toyoshima F, et al. Prospective randomized controlled trial to compare the effects of omeprazole and famotidine in preventing delayed bleeding and promoting ulcer healing after endoscopic submucosal dissection. J Gastroenterol Hepatol. 2012;27(9):1441–6. doi: 10.1111/j.1440-1746.2012.07144.x 22497427

[29] Kawano S, Okada H, Kawahara Y, Hori K, Tanioka D, Tsuzuki T, et al. Proton pump inhibitor dose-related healing rate of artificial ulcers after endoscopic submucosal dissection: A prospective randomized controlled trial. Digestion. 2011;84(1):46–53. doi: 10.1159/000321660 21304241

[30] Ichida T, Ueyama S, Eto T, Kusano F, Sakai Y. Randomized controlled trial comparing the effects of vonoprazan plus rebamipide and esomeprazole plus rebamipide on gastric ulcer healing induced by endoscopic submucosal dissection. Intern Med. 2019;58(2):159–66. doi: 10.2169/internalmedicine.1146-18 30210115 PMC6378166

[31] Lee JY, Jang JS. The effect of PPIs Alone, PPIs plus cytoprotective agent, and H2RA plus cytoprotective agent on ulcer healing after endoscopic submucosal dissection: A prospective randomized controlled trial. Arab J Gastroenterol. 2021;22(2):115–20. doi: 10.1016/j.ajg.2020.09.007 33551349

[32] Ahn JY, Choi CH, Lee JW, Park SJ, Kim JW, Chang SK, et al. The effect of sequential therapy with lansoprazole and ecabet sodium in treating iatrogenic gastric ulcer after endoscopic submucosal dissection: A randomized prospective study. J Dig Dis. 2015;16(2):75–82. doi: 10.1111/1751-2980.12214 25420889

[33] Kim BW, Park JJ, Moon HS, Lee WS, Shim KN, Baik GH, et al. The effect of tegoprazan on the treatment of endoscopic resection-induced artificial ulcers: A multicenter, randomized, active-controlled study. Gut Liver. 2024;18(2):257–64. doi: 10.5009/gnl230242 38384180 PMC10938149

[34] Kato M, Hosoe N, Gotoda T, Kusano C, Kuramochi M, Yoshida H, et al. Treatment with vonoprazan for 3 weeks is not inferior to 8 weeks for the management of gastric ESD: A multicenter noninferiority randomized study. J Gastroenterol. 2023, 58(4):358–66. 10.1007/s00535-023-01966-z 36781490

[35] Arai M, Matsumura T, Okimoto K, Oyamada A, Saito K, Minemura S, et al. Two-week treatment with proton pump inhibitor is sufficient for healing post endoscopic submucosal dissection ulcers. World J Gastroenterol. 2014;20(43):16318–22. doi: 10.3748/wjg.v20.i43.16318 25473190 PMC4239524

[36] Takahashi K, Sato Y, Kohisa J, Watanabe J, Sato H, Mizuno K, et al. Vonoprazan 20 mg vs lansoprazole 30 mg for endoscopic submucosal dissection-induced gastric ulcers. World J Gastrointest Endosc. 2016;8(19):716–22. doi: 10.4253/wjge.v8.i19.716 27909552 PMC5114461

[37] Shibata H, Fujiyoshi T, Kawata N, Obayashi T, Esaki M, Kikuchi M, et al. Vonoprazan or proton pump inhibitor for gastric endoscopic submucosal dissection in patients taking antithrombotic agents. J Gastroenterol Hepatol. 2025;40(8):1890–8. doi: 10.1111/jgh.17027 40420803 PMC12315787

[38] Yang YJ, Lim H, Lee JG, Oh CK, Kim TW, Lee SP, et al. Comparison of rebamipide and tegoprazan combination therapy and tegoprazan monotherapy for ESD induced gastric ulcers a randomized multicenter study. Sci Rep. 2025;15(1):27127. doi: 10.1038/s41598-025-11964-1 40715454 PMC12297290

[39] Kobayashi M, Takeuchi M, Hashimoto S, Mizuno K, Sato Y, Narisawa R, et al. Contributing factors to gastric ulcer healing after endoscopic submucosal dissection including the promoting effect of rebamipide. Dig Dis Sci. 2012;57(1):119–26. doi: 10.1007/s10620-011-1850-4 21842241

[40] Nakamura K, Ihara E, Akiho H, Akahoshi K, Harada N, Ochiai T, et al. Limited effect of rebamipide in addition to proton pump inhibitor (PPI) in the treatment of post-endoscopic submucosal dissection gastric ulcers: A randomized controlled trial comparing PPI plus rebamipide combination therapy with PPI monotherapy. Gut Liver. 2016;10(6):917–24. doi: 10.5009/gnl15486 27282261 PMC5087931

[41] Chen L, Jiang D, Hu D, Cui X. Comparison of vonoprazan and proton pump inhibitors for the treatment of gastric endoscopic submucosal dissection-induced ulcer: An updated systematic review and meta-analysis. BMC Gastroenterol. 2024;24(1):110. doi: 10.1186/s12876-024-03198-8 38491413 PMC10943859

[42] Wang J, Guo X, Ye C, Yu S, Zhang J, Song J, et al. Efficacy and safety of proton pump inhibitors (PPIs) plus rebamipide for endoscopic submucosal dissection-induced ulcers: A meta-analysis. Intern Med. 2014;53(12):1243–8. doi: 10.2169/internalmedicine.53.2160 24930641

[43] Nishizawa T, Suzuki H, Kanai T, Yahagi N. Proton pump inhibitor alone vs proton pump inhibitor plus mucosal protective agents for endoscopic submucosal dissection-induced ulcer: A systematic review and meta-analysis. J Clin Biochem Nutr. 2015;56(2):85–90. doi: 10.3164/jcbn.14-101 25759512 PMC4345174

[44] Eutamene H, Beaufrand C, Harkat C, Theodorou V. The role of mucoprotectants in the management of gastrointestinal disorders. Expert Rev Gastroenterol Hepatol. 2018;12(1):83–90. doi: 10.1080/17474124.2018.1378573 28946778

[45] Xu K, Li J, Guo S, Zhang J, Zhang X. Gastric mucosal repair by Men’s Huwei Powder via EGF-NO/PGE2-PI3K-TLR4 in RELISH: Restoring Equilibrium through long-term integration of synergistic health. Front Pharmacol. 2025;16:1594089. doi: 10.3389/fphar.2025.1594089 40717975 PMC12289676

[46] Maruoka D, Arai M, Kasamatsu S, Ishigami H, Taida T, Okimoto K, et al. Vonoprazan is superior to proton pump inhibitors in healing artificial ulcers of the stomach post-endoscopic submucosal dissection: A propensity score-matching analysis. Dig Endosc. 2017;29(1):57–64. doi: 10.1111/den.12705 27492962

