# Supplementary material for: Time-dependent efficacy of seven pharmacological regimens for ulcer healing and delayed bleeding prevention after gastric endoscopic submucosal dissection: A frequentist network meta-analysis
Source: PLoS One. 2026 Nov 10;21(11):e0362119. doi: 10.1371/journal.pone.0362119 (PMC13750581; doi:10.1371/journal.pone.0362119)
Supplement: Supplementary file 1 [file pone.0362119.s001.docx]

| **Section and Topic** | **Item #** | **Checklist item** | **Location where item is reported** |
| --- | --- | --- | --- |
| **TITLE** | | |  |
| Title | 1 | Identify the report as a systematic review. | Title page (Manuscript Title) |
| **ABSTRACT** | | |  |
| Abstract | 2 | See the PRISMA 2020 for Abstracts checklist. | Abstract section (includes Objective, Methods, Results, Conclusions consistent with PRISMA 2020 Abstract guidelines) |
| **INTRODUCTION** | | |  |
| Rationale | 3 | Describe the rationale for the review in the context of existing knowledge. | 1. Introduction section (discusses unmet clinical needs in post-ESD ulcer management and limitations of existing evidence) |
| Objectives | 4 | Provide an explicit statement of the objective(s) or question(s) the review addresses. | Abstract (Objective part); 1. Introduction (final paragraph, research aim of the network meta-analysis) |
| **METHODS** | | |  |
| Eligibility criteria | 5 | Specify the inclusion and exclusion criteria for the review and how studies were grouped for the syntheses. | 2. Materials and Methods - Eligibility criteria subsection (types of studies, participants, interventions; studies grouped by 7 pharmacological regimens for synthesis) |
| Information sources | 6 | Specify all databases, registers, websites, organisations, reference lists and other sources searched or consulted to identify studies. Specify the date when each source was last searched or consulted. | 2. Materials and Methods - Search strategy subsection (databases: PubMed/MEDLINE, Embase, CENTRAL; search cut-off date: Dec 10th 2025; no other sources reported) |
| Search strategy | 7 | Present the full search strategies for all databases, registers and websites, including any filters and limits used. | 2. Materials and Methods - Search strategy subsection (full MeSH + free-text search formula provided; randomized trial filter applied) |
| Selection process | 8 | Specify the methods used to decide whether a study met the inclusion criteria of the review, including how many reviewers screened each record and each report retrieved, whether they worked independently, and if applicable, details of automation tools used in the process. | 2. Materials and Methods - Study selection subsection (two independent reviewers for title/abstract screening and full-text assessment; third reviewer for disagreement resolution; no automation tools used) |
| Data collection process | 9 | Specify the methods used to collect data from reports, including how many reviewers collected data from each report, whether they worked independently, any processes for obtaining or confirming data from study investigators, and if applicable, details of automation tools used in the process. | 2. Materials and Methods - Data extraction subsection (two independent reviewers for data extraction; third reviewer for discrepancy resolution; no investigator data confirmation/automation tools used) |
| Data items | 10a | List and define all outcomes for which data were sought. Specify whether all results that were compatible with each outcome domain in each study were sought (e.g. for all measures, time points, analyses), and if not, the methods used to decide which results to collect. | 2. Materials and Methods - Outcomes subsection (primary outcomes: delayed bleeding/perforation, ulcer healing (Sakita-Miwa classification S1/2); 4/8-week time points extracted where available; only pre-specified time points/measures collected) |
|  | 10b | List and define all other variables for which data were sought (e.g. participant and intervention characteristics, funding sources). Describe any assumptions made about any missing or unclear information. | 2. Materials and Methods - Data extraction subsection (variables: study identifiers, design, sample size, follow-up duration; Table 1 (studyID, year, region, treatment regimens); no assumptions for missing/unclear information reported) |
| Study risk of bias assessment | 11 | Specify the methods used to assess risk of bias in the included studies, including details of the tool(s) used, how many reviewers assessed each study and whether they worked independently, and if applicable, details of automation tools used in the process. | 2. Materials and Methods - Risk of Bias Assessment subsection (Cochrane RoB tool (7 domains); two independent reviewers; third reviewer for adjudication; Figure 2 (risk of bias graph); no automation tools used) |
| Effect measures | 12 | Specify for each outcome the effect measure(s) (e.g. risk ratio, mean difference) used in the synthesis or presentation of results. | 2. Materials and Methods - Data synthesis and statistical analysis subsection (binary outcomes: OR, RR; continuous outcomes: MD; 95% CIs for all; Table 2 (all effect measures reported)) |
| Synthesis methods | 13a | Describe the processes used to decide which studies were eligible for each synthesis (e.g. tabulating the study intervention characteristics and comparing against the planned groups for each synthesis (item #5)). | 2. Materials and Methods - Data synthesis and statistical analysis subsection; 3. Results - 3.1 Study Inclusion and Network Structures (studies grouped by 7 pharmacological regimens; full network connectivity confirmed for synthesis) |
|  | 13b | Describe any methods required to prepare the data for presentation or synthesis, such as handling of missing summary statistics, or data conversions. | 2. Materials and Methods - Data synthesis and statistical analysis subsection (medians/IQRs approximated to means/SDs for continuous data; no other data preparation methods reported) |
|  | 13c | Describe any methods used to tabulate or visually display results of individual studies and syntheses. | 2. Materials and Methods - Data synthesis and statistical analysis subsection (network geometry (Figure3), P-score rankings (Figures4.1-4.4), Table1 (individual studies), Table2 (synthesized results)) |
|  | 13d | Describe any methods used to synthesize results and provide a rationale for the choice(s). If meta-analysis was performed, describe the model(s), method(s) to identify the presence and extent of statistical heterogeneity, and software package(s) used. | 2. Materials and Methods - Data synthesis and statistical analysis subsection (frequentist network meta-analysis; random-effects model (for heterogeneity); heterogeneity assessed via I², τ²; inconsistency via design-by-treatment interaction/node-splitting; software: R 4.3 (netmeta/mvmeta), Stata (network commands)) |
|  | 13e | Describe any methods used to explore possible causes of heterogeneity among study results (e.g. subgroup analysis, meta-regression). | 2. Materials and Methods - Data synthesis and statistical analysis subsection; 3. Results (3.2-3.4) (subgroup analysis by sample size; 3.5 Heterogeneity (quantitative heterogeneity analysis for all outcomes)) |
|  | 13f | Describe any sensitivity analyses conducted to assess robustness of the synthesized results. | 2. Materials and Methods - Data synthesis and statistical analysis subsection; 3. Results (3.2-3.4) (fixed vs. random effects model, leave-one-out analysis, exclusion of high-risk bias/imputed data studies) |
| Reporting bias assessment | 14 | Describe any methods used to assess risk of bias due to missing results in a synthesis (arising from reporting biases). | 2. Materials and Methods - Data synthesis and statistical analysis subsection; 3. Results (3.2-3.4) (Egger’s regression, Begg’s test, trim-and-fill analysis, funnel plots for publication bias assessment) |
| Certainty assessment | 15 | Describe any methods used to assess certainty (or confidence) in the body of evidence for an outcome. | 2. Materials and Methods - Data synthesis and statistical analysis: - Tool: GRADE (Grading of Recommendations, Assessment, Development and Evaluations) |
| **RESULTS** | | |  |
| Study selection | 16a | Describe the results of the search and selection process, from the number of records identified in the search to the number of studies included in the review, ideally using a flow diagram. | 2. Materials and Methods - Study selection subsection; Figure1 (PRISMA flow diagram with full numerical details of search/selection); 3.1 Study Inclusion and Network Structures (30 RCTs included) |
|  | 16b | Cite studies that might appear to meet the inclusion criteria, but which were excluded, and explain why they were excluded. | 2. Materials and Methods - Study selection subsection; Figure1 (exclusion reasons: non-ESD, non-standard RCT, review/letter/editorial, lack of prognosis results, no required treatment strategy); no individual excluded studies cited |
| Study characteristics | 17 | Cite each included study and present its characteristics. | 3.1 Study Inclusion and Network Structures; Table1 (all 30 included RCTs with studyID, year, region, cases, treatment regimens) |
| Risk of bias in studies | 18 | Present assessments of risk of bias for each included study. | 2. Materials and Methods - Risk of Bias Assessment subsection; Figure2 (risk of bias graph (percentages for each RoB domain across all studies); narrative/tabular results mentioned, no individual study RoB table reported) |
| Results of individual studies | 19 | For all outcomes, present, for each study: (a) summary statistics for each group (where appropriate) and (b) an effect estimate and its precision (e.g. confidence/credible interval), ideally using structured tables or plots. | 3. Results (3.2-3.4); Table1 (study-level treatment regimens/cases); Table2 (pooled effect estimates/95% CIs for all outcomes; individual study summary statistics integrated into pooled analyses) |
| Results of syntheses | 20a | For each synthesis, briefly summarise the characteristics and risk of bias among contributing studies. | 3. Results (3.2-3.4) (each outcome synthesis includes study count/pairwise comparisons; 3.5 Heterogeneity (bias/heterogeneity summary for all syntheses); Figure2 (overall RoB for contributing studies)) |
|  | 20b | Present results of all statistical syntheses conducted. If meta-analysis was done, present for each the summary estimate and its precision (e.g. confidence/credible interval) and measures of statistical heterogeneity. If comparing groups, describe the direction of the effect. | 3. Results (3.2-3.5); Table2 (all pooled OR/MD/RR, 95% CIs, P-values; P-scores for ranking); 3.5 Heterogeneity (I², τ², P-values for all outcomes; direction of effects described in text for each regimen vs. PPI monotherapy) |
|  | 20c | Present results of all investigations of possible causes of heterogeneity among study results. | 3.5 Heterogeneity (quantitative heterogeneity results for all outcomes); 3. Results (3.2-3.4) (subgroup analysis by sample size for all outcomes; heterogeneity discussion in text) |
|  | 20d | Present results of all sensitivity analyses conducted to assess the robustness of the synthesized results. | 3. Results (3.2-3.4) (sensitivity analysis results for each outcome: fixed/random effects, leave-one-out, subgroup analysis; all results reported in text with effect estimates/P-values) |
| Reporting biases | 21 | Present assessments of risk of bias due to missing results (arising from reporting biases) for each synthesis assessed. | 3. Results (3.2-3.4) (publication bias results for each outcome: Egger/Begg test P-values, trim-and-fill analysis, funnel plot interpretation; all reported in text) |
| Certainty of evidence | 22 | Present assessments of certainty (or confidence) in the body of evidence for each outcome assessed. | Resrults3.6 certainty of evidence |
| **DISCUSSION** | | |  |
| Discussion | 23a | Provide a general interpretation of the results in the context of other evidence. | 4. Discussion section (core findings linked to existing ESD/ulcer healing literature; comparison with previous meta-analyses) |
|  | 23b | Discuss any limitations of the evidence included in the review. | 4. Discussion section (limitations: uneven sample size across regimens, severe heterogeneity in 8-week healing, limited direct RCT evidence for some treatment pairs) |
|  | 23c | Discuss any limitations of the review processes used. | 4. Discussion section (limitations: publication bias in 4-week healing, indirect comparisons for some regimens, no individual study RoB details) |
|  | 23d | Discuss implications of the results for practice, policy, and future research. | 4. Discussion section (clinical practice: individualized post-ESD ulcer management; future research: large-sample RCTs for H2RA/combination regimens); |
| **OTHER INFORMATION** | | |  |
| Registration and protocol | 24a | Provide registration information for the review, including register name and registration number, or state that the review was not registered. | 2. Materials and Methods - 2.1 Study design and registration (PROSPERO database; registration number: CRD420251057886) |
|  | 24b | Indicate where the review protocol can be accessed, or state that a protocol was not prepared. | 2. Materials and Methods - 2.1 Study design and registration (protocol prospectively registered in PROSPERO; available via PROSPERO database with the registration number) |
|  | 24c | Describe and explain any amendments to information provided at registration or in the protocol. | No amendments reported |
| Support | 25 | Describe sources of financial or non-financial support for the review, and the role of the funders or sponsors in the review. | Title page (Funding section) (no financial or non-financial support received) |
| Competing interests | 26 | Declare any competing interests of review authors. | Title page no competing interests |
| Availability of data, code and other materials | 27 | Report which of the following are publicly available and where they can be found: template data collection forms; data extracted from included studies; data used for all analyses; analytic code; any other materials used in the review. | 2. Materials and Methods - Data synthesis and statistical analysis subsection (all analytic code, extracted data available from the corresponding author upon reasonable request; no public repository reported for template forms/other materials) |

*From:* Page MJ, McKenzie JE, Bossuyt PM, Boutron I, Hoffmann TC, Mulrow CD, et al. The PRISMA 2020 statement: an updated guideline for reporting systematic reviews. BMJ 2021;372:n71. doi: 10.1136/bmj.n71. This work is licensed under CC BY 4.0. To view a copy of this license, visit <https://creativecommons.org/licenses/by/4.0/>
