# Supplementary material for: Time-dependent efficacy of seven pharmacological regimens for ulcer healing and delayed bleeding prevention after gastric endoscopic submucosal dissection: A frequentist network meta-analysis
Source: PLoS One. 2026 Nov 10;21(11):e0362119. doi: 10.1371/journal.pone.0362119 (PMC13750581; doi:10.1371/journal.pone.0362119)
Supplement: Supplementary file 2 [file pone.0362119.s002.docx]

**PRISMA 2020 Expanded Checklist**

| Section and Topic | Item # | Items and Elements Recommended for Reporting | Corresponding Manuscript Location & Details |
| --- | --- | --- | --- |
| **TITLE** | 1 | Identify the report as a systematic review; provide informative title (population, intervention, objective); optional: method/study design. | • Title page: Explicitly labeled "A Frequentist Network Meta-Analysis"; title includes core elements: "Seven Pharmacological Regimens for Ulcer Healing and Delayed Bleeding Prevention After Endoscopic Submucosal Dissection" |
| **ABSTRACT** | 2 | Address each item in PRISMA 2020 for Abstracts checklist (Objective, Methods, Results, Conclusions). | • Abstract section: Clearly presents Objective (evaluate 7 regimens), Methods (frequentist NMA, RCTs up to 2025), Results (key OR/RR, P-scores), Conclusions (time-dependent efficacy) |
| **INTRODUCTION - RATIONALE** | 3 | Describe current knowledge/uncertainties; explain review importance; cite existing similar reviews if applicable. | • 1. Introduction: Discusses ESD-induced "artificial ulcers" (4-12% delayed bleeding risk), limitations of current clinical recommendations (no consensus on P-CAB/PPIs/combinations), and need for focused evidence synthesis |
| **INTRODUCTION - OBJECTIVES** | 4 | Explicit objective/question using PICO-like framework; specify comparisons. | • Abstract (Objective) + 1. Introduction (final paragraph): "Evaluate and rank the efficacy of 7 pharmacological regimens for ulcer healing and delayed bleeding prevention after ESD" : Population (ESD patients), Intervention (7 regimens), Comparator (PPI monotherapy as reference), Outcome (4/8-week ulcer healing, delayed bleeding/perforation) |
| **METHODS - ELIGIBILITY CRITERIA** | 5 | Specify PICO components, study design, setting, follow-up; report eligibility restrictions (language/year); define synthesis groups. | • 2. Materials and Methods - Eligibility criteria: - Study type: RCTs (exclude non-RCTs, case reports, reviews, abstracts without full data) Participants: Adults (≥18 years) with gastric/esophageal ESD - Interventions: 7 regimens (PPI monotherapy, P-CAB monotherapy, etc.) - Outcomes: Delayed bleeding/perforation, ulcer healing (Sakita-Miwa S1/2) at 4/8 weeks - Synthesis groups: Grouped by 7 regimens (Regimen 1-7) |
| **METHODS - INFORMATION SOURCES** | 6 | Specify databases/registers/websites; report last search date; note other sources (reference lists/organizations). | • 2. Materials and Methods - Search strategy: - Databases: PubMed/MEDLINE, Embase, CENTRAL - Search date: Dec 10th 2025 - Other sources: None (no reference list screening/organization contacts reported) |
| **METHODS - SEARCH STRATEGY** | 7 | Provide full search strategy for each database; describe filters/limits; note adaptations/validation/peer review. | • 2. Materials and Methods - Search strategy，etc. |
| **METHODS - SELECTION PROCESS** | 8 | Report number of reviewers (screening/data extraction), independence, disagreement resolution; note automation tools/translation. | • 2. Materials and Methods - Study selection: - Two independent reviewers Disagreement resolution: Discussion → third reviewer consultation - Automation tools: None - Translation: Not needed (no non-English studies included) |
| **METHODS - DATA COLLECTION PROCESS** | 9 | Report number of reviewers, independence, disagreement resolution; note automation tools/translation/data confirmation. | • 2. Materials and Methods - Data extraction: - Two independent reviewers (data extraction) - Disagreement resolution: Discussion → third reviewer adjudication - Automation tools: None - Data confirmation: No contact with study investigators reported - Translation: Not needed |
| **METHODS - DATA ITEMS (OUTCOMES)** | 10a | List/define outcome domains/time points; specify if all results were sought; note changes to outcomes. | • 2. Materials and Methods - Outcomes: - Primary outcomes: (1) Delayed bleeding/perforation (definition provided), (2) Ulcer healing (Sakita-Miwa S1/2) - Time points: 4 and 8 weeks (extracted where available) - Result selection: Pre-specified time points (4/8 weeks) only - Changes to outcomes: None reported |
| **METHODS - DATA ITEMS (OTHER VARIABLES)** | 10b | List/define other variables; describe assumptions for missing/unclear data. | • 2. Materials and Methods - Data extraction: - Variables: Study identifiers (author, year, region), sample size, intervention details, follow-up duration - Missing data assumptions: None reported - Tool for data items: Standardized extraction form (piloted) |
| **METHODS - STUDY RISK OF BIAS ASSESSMENT** | 11 | Specify tool/version, domains, overall judgment rules; report reviewer number/independence; note adaptations/automation. | • 2. Materials and Methods - Risk of Bias Assessment: - Tool: Cochrane RoB tool (Review Manager 5.4), 7 domains (random sequence generation, allocation concealment, etc.) - Judgment: Low/unclear/high risk; overall judgment per outcome - Reviewers: Two independent reviewers; disagreement resolved via discussion/third reviewer - Adaptations: None; automation tools: None |
| **METHODS - EFFECT MEASURES** | 12 | Specify effect measures per outcome type; note interpretation thresholds; justify choices. | • 2. Materials and Methods - Data synthesis and statistical analysis: - Binary outcomes (bleeding/healing): Odds Ratio (OR), Relative Risk (RR) with 95%CI - Continuous outcomes (ulcer reduction rate): Mean Difference (MD) with 95%CI - Thresholds: Statistical significance (P<0.05); no clinical significance thresholds reported - Justification: Standard effect measures for NMA of RCTs |
| **METHODS - SYNTHESIS METHODS (ELIGIBILITY)** | 13a | Describe process for selecting studies for each synthesis; link to objectives. | • 3. Results - 3.1 Study Inclusion and Network Structures: - Process: Studies eligible for synthesis if they reported the outcome (4/8-week healing, bleeding) and compared ≥1 of the 7 regimens - Link to objectives: Syntheses aligned with "rank efficacy of 7 regimens" (all regimens included in full network) |
| **METHODS - SYNTHESIS METHODS (PREPARING)** | 13b | Describe data preparation (missing statistics, conversions). | • 2. Materials and Methods - Data synthesis and statistical analysis: - Conversions: Medians + interquartile ranges (IQR) approximated to means + standard deviations (SD) for continuous outcomes - Missing summary statistics: No specific method reported (assumed complete data from included RCTs) |
| **METHODS - SYNTHESIS METHODS (TABULATION)** | 13c | Describe tabular/graphical methods; note ordering/grouping rationale. | • 2. Materials and Methods - Data synthesis and statistical analysis + Results: - Tabular: Table1 (study characteristics), Table2 (comparative efficacy) - Graphical: PRISMA flow diagram (Figure1), risk of bias graph (Figure2), network geometry (Figure3), P-score ranking plots (Figure4.1-4.4) - Ordering: Studies ordered by year in Table1; regimens ordered by P-score in Figure4 |
| **METHODS - SYNTHESIS METHODS (STATISTICAL)** | 13d | Describe synthesis method/rationale; if meta-analysis: model, heterogeneity measures, software. | • 2. Materials and Methods - Data synthesis and statistical analysis: - Synthesis method: Frequentist network meta-analysis (preserves randomization) - Rationale: Enables simultaneous comparison of 7 regimens (direct + indirect evidence) - Meta-analysis model: Random-effects model (accounts for between-study heterogeneity) - Heterogeneity measures: I² statistic, tau-squared (τ²) - Software: R 4.3 (netmeta/mvmeta packages) or Stata (network commands) |
| **METHODS - SYNTHESIS METHODS (HETEROGENEITY)** | 13e | Describe methods to explore heterogeneity (subgroup/meta-regression); specify factors/levels. | • 2. Materials and Methods - Data synthesis and statistical analysis + 3. Results (3.2-3.4): - Method: Subgroup analysis (by sample size: median=138, large-sample ≥138) - Factors: Sample size (explore if small-sample studies drive results) - Pre-specified: Yes (part of sensitivity analysis plan) |
| **METHODS - SYNTHESIS METHODS (SENSITIVITY)** | 13f | Describe sensitivity analyses; note pre-specified vs. post-hoc. | • 2. Materials and Methods - Data synthesis and statistical analysis + 3. Results (3.2-3.4): - Analyses: (1) Fixed vs. random effects model comparison; (2) Leave-one-out analysis; (3) Exclude high-risk bias/imputed data studies - Pre-specified: Yes (all part of protocol) |
| **METHODS - REPORTING BIAS ASSESSMENT** | 14 | Specify methods (tool/graphs/tests); report reviewer number/independence; note adaptations. | • 2. Materials and Methods - Data synthesis and statistical analysis + 3. Results (3.2-3.4): - Methods: Egger’s regression test, Begg’s test, trim-and-fill analysis, funnel plots - Reviewers: Implied (same as data extraction/risk of bias reviewers) - Independence: Yes (consistent with other assessments) - Adaptations: None |
| **METHODS - CERTAINTY ASSESSMENT** | 15 | Specify tool/version, factors/criteria, decision rules; report reviewer number/independence. | • 2. Materials and Methods - Data synthesis and statistical analysis: - Tool: GRADE (Grading of Recommendations, Assessment, Development and Evaluations) |
| **RESULTS - STUDY SELECTION (FLOW)** | 16a | Report search/selection numbers via flow diagram; note update/automation. | • Figure1 (PRISMA Flow Diagram) + 2. Materials and Methods - Study selection: - Numbers: Identified (99+17=116) → duplicates removed (18) → screened (98) → excluded (28) → full-text assessed (33) → excluded (3) → included (30 RCTs) - Automation: None - Update: Not an update (first review) |
| **RESULTS - STUDY SELECTION (EXCLUDED)** | 16b | Cite excluded studies that appear eligible; explain reasons. | • Figure1: Exclusion reasons (1) Not ESD (n=8); (2) Non-standard RCT (n=11); (3) Review/letter/editorial (n=9); (4) Lack of prognosis data (n=2); (5) No required treatment strategy (n=1) - Citation of excluded studies: None (only aggregate reasons reported) |
| **RESULTS - STUDY CHARACTERISTICS** | 17 | Cite all included studies; present key characteristics in tables/figures; summarize interventions. | • Table1 (Characteristics of Included RCTs): - Cites 30 studies (studyID, year, region, sample size, treatment regimens) - Key characteristics: All RCTs (Japan/Korea/China), 7 regimens, sample size range 20-242 patients - Intervention summary: Clear grouping of 7 regimens (Regimen 1-7) |
| **RESULTS - RISK OF BIAS IN STUDIES** | 18 | Present domain-specific/overall risk of bias in tables/figures; justify judgments. | • Figure2 (Risk of Bias Graph) + 2. Materials and Methods - Risk of Bias Assessment: - Graph: Percentages of studies with low/unclear/high risk per domain (e.g., allocation concealment: high/unclear risk) - Justification: Narrative summary (no individual study RoB table, but "detailed results in tabular and narrative forms" reported) |
| **RESULTS - RESULTS OF INDIVIDUAL STUDIES** | 19 | Present group-level summary stats + effect estimates/precision for each study; note data source. | • Table1 + Table2: - Group-level stats: Sample size per arm (Table1), event counts (implied in OR/RR calculations) - Effect estimates: Pooled OR/MD/RR (Table2); individual study estimates integrated into pooled analyses (not reported separately) - Data source: Full-text RCTs (no abstracts included) |
| **RESULTS - RESULTS OF SYNTHESES (CHARACTERISTICS)** | 20a | Summarize study characteristics/risk of bias for each synthesis; list included studies. | • 3. Results (3.2-3.4): - For each outcome: Number of studies/pairwise comparisons (e.g., 4-week healing: 18 studies, 16 pairwise comparisons) - Risk of bias summary: Mild/severe heterogeneity (e.g., 4-week healing: I²=32.5%, mild heterogeneity) - Included studies: Implied (linked to Table1) |
| **RESULTS - RESULTS OF SYNTHESES (STATISTICAL)** | 20b | Present pooled estimates/precision, heterogeneity measures; describe effect direction. | • Table2 + 3. Results (3.2-3.4): - Pooled estimates: OR/MD/RR + 95%CI + P-values (e.g., 4-week healing: P-CAB monotherapy OR=1.57, 95%CI=1.05-2.36, P=0.029) - Heterogeneity: I², τ², P-values (e.g., 8-week healing: I²=89.6%, τ²=1.9133, P<0.0001) - Effect direction: Superiority/inferiority vs. PPI monotherapy (e.g., P-CAB+MP superior for 8-week healing) |
| **RESULTS - RESULTS OF SYNTHESES (HETEROGENEITY)** | 20c | Present subgroup/meta-regression results; note study contribution. | • 3. Results (3.2-3.4) Subgroup analysis: - Sample size subgroup: Large-sample studies (≥138) confirm P-CAB monotherapy superiority (4-week healing) but not PPI+MP - No significant effect modification reported - Study contribution: No single study dominates (leave-one-out analysis confirms) |
| **RESULTS - RESULTS OF SYNTHESES (SENSITIVITY)** | 20d | Present sensitivity analysis results; comment on robustness. | • 3. Results (3.2-3.4) Sensitivity Analysis: - Fixed vs. random effects: Minimal OR/MD differences (<6%), significance unchanged - Leave-one-out: OR/MD ranges narrow (e.g., P-CAB monotherapy OR=1.492-1.658), all P<0.05 - Robustness: Key findings (P-CAB+MP for 8-week healing) stable |
| **RESULTS - REPORTING BIASSES** | 21 | Present reporting bias assessments (tool responses/funnel plots/tests); note sensitivity analyses for missing results. | • 3. Results (3.2-3.4) Publication Bias Assessment: - Funnel plots: No obvious asymmetry (8-week healing) or potential bias (4-week healing) - Tests: Egger’s (4-week healing P=0.014) + Begg’s (P=0.0269) suggest potential bias; trim-and-fill adjusts OR to 0.5638 but core conclusion unchanged - Missing results: Trim-and-fill estimates 2 missing studies (4-week healing) |
| **RESULTS - CERTAINTY OF EVIDENCE** | 22 | Report overall certainty per outcome; explain rating up/down; use Summary of Findings tables. | • 3.6 Certainty of evidence: - 4-week mucosal healing: Moderate (unclear bias + mild heterogeneity) ,etc. |
| **DISCUSSION - INTERPRETATION** | 23a | Interpret results in context of other evidence; link to objectives. | • 4. Discussion: - Context: Compares findings to previous NMA (e.g., Gao et al. 2020) and RCTs (e.g., Tsuchiya et al. 2017) - Alignment with objectives: Confirms time-dependent efficacy (short-term: P-CAB/PPI+MP; long-term: P-CAB+MP) |
| **DISCUSSION - LIMITATIONS OF EVIDENCE** | 23b | Discuss evidence limitations (study quality, heterogeneity, generalizability). | • 4. Discussion - Limitations: - Uneven sample size (H2RA evaluated in 3-5 studies) - Severe heterogeneity (8-week healing: I²=89.6%) - Generalizability: Studies from East Asia (Japan/Korea/China) only |
| **DISCUSSION - LIMITATIONS OF REVIEW PROCESSES** | 23c | Discuss review method limitations; comment on impact. | • 4. Discussion - Limitations: - Indirect comparisons for some treatment pairs (no direct RCTs) - Publication bias (4-week healing) - No individual patient data (cannot explore H. pylori/subgroup effects) - Impact: Limited by indirect evidence but inconsistency tests (P>0.05) support reliability |
| **DISCUSSION - IMPLICATIONS** | 23d | Discuss practice/policy implications; make future research recommendations. | • 4. Discussion + 5. Conclusion: - Practice: Individualized care (short-term: P-CAB/PPI+MP; long-term: P-CAB+MP; bleeding prevention: PPI) - Future research: Large-sample RCTs for H2RA/combination regimens, global studies, individual patient data meta-analysis |
| **OTHER INFORMATION - REGISTRATION** | 24a | Provide register name + registration number; or state unregistered. | • 2. Materials and Methods - 2.1 Study design and registration: - Register: PROSPERO - Registration number: CRD420251057886 |
| **OTHER INFORMATION - PROTOCOL** | 24b | Indicate protocol access (citation/DOI/link); or state no protocol. | • 2. Materials and Methods - 2.1 Study design and registration: - Protocol access: Prospector registered (available via PROSPERO with registration number) |
| **OTHER INFORMATION - AMENDMENTS** | 24c | Describe protocol amendments (what/why/when); or state no amendments. | • 2. Materials and Methods - 2.1 Study design and registration: - No protocol amendments reported |
| **OTHER INFORMATION - SUPPORT** | 25 | Describe financial/non-financial support; report funder role. | • Title page - Funding: - No financial or non-financial support received - Funder role: None |
| **OTHER INFORMATION - COMPETING INTERESTS** | 26 | Disclose author competing interests; report management if applicable. | • Title page - Competing interest: - The authors declared no competing interests |
| **OTHER INFORMATION - AVAILABILITY OF DATA/CODE** | 27 | Report public availability of data/forms/code; note access details. | • 2. Materials and Methods - Data synthesis and statistical analysis: - Available: Analytic code, extracted data (upon reasonable request to corresponding author) - Not available: Template data collection forms (no public repository reported) |
