# Supplementary material for: Time-dependent efficacy of seven pharmacological regimens for ulcer healing and delayed bleeding prevention after gastric endoscopic submucosal dissection: A frequentist network meta-analysis
Source: PLoS One. 2026 Nov 10;21(11):e0362119. doi: 10.1371/journal.pone.0362119 (PMC13750581; doi:10.1371/journal.pone.0362119)
Supplement: Supplementary file 3 [file pone.0362119.s003.docx]

| **Comparison Dimension** | **PMID: 41442424 (Chen et al., 2025, *Pharmacology*)** | **Present Study** |
| --- | --- | --- |
| **1. Search period and literature cutoff** | Searched PubMed, Embase, Cochrane Library, Web of Science, ClinicalTrials.gov up to **January 2025** | Searched PubMed/MEDLINE, Embase, CENTRAL up to **December 10, 2025** (11 months more recent literature) |
| **2. Number and type of included studies** | **20** studies; all studies from East Asia (Japan, Korea, China); no explicit exclusion of conference abstracts or animal studies stated | **31 RCTs**; explicitly excluded non-randomized studies, conference abstracts without complete raw data, and animal studies; all full-text publications |
| **3. Intervention nodes and treatment classification** | **10 treatment arms**, all focused on **PPI combined with different mucosal protectants** (rebamipide, ecabet, polaprezinc, teprenone, bismuth magnesium granules, hydrotalcite, etc.) plus PPI monotherapy; **no P-CAB monotherapy, no H2RA-containing regimens, no mucosal protector monotherapy** | **7 treatment arms** covering mainstream clinical regimens: PPI, P-CAB, H2RA, mucosal protector (MP) monotherapy, PPI+MP, P-CAB+MP, H2RA+MP; includes acid-suppressant monotherapy comparisons absent in the prior study |
| **4. 4-week ulcer healing outcomes** | Bismuth magnesium granules + PPI ranked first for ulcer area reduction (MD −62.50 mm², 95%CI −87.25 to −37.75; SUCRA 94%); multiple combination regimens showed statistically significant superiority over PPI monotherapy | P-CAB monotherapy ranked first (OR 1.61, 95%CI 0.96–2.68, P=0.069); **no regimen achieved statistically significant superiority** over PPI; combination regimens showed only numerically favorable trends |
| **5. 8-week ulcer healing outcomes** | Ecabet + PPI achieved highest healing rate (RR 7.71, 95%CI 5.52–9.91); several combinations significantly superior to PPI | PPI+MP ranked highest (OR 1.29, 95%CI 0.71–2.37, P=0.405); **no statistically significant differences** between any regimen and PPI |
| **6. Safety outcomes (delayed bleeding/perforation)** | Teprenone + PPI ranked best for bleeding prevention (RR −3.41, 95%CI −5.43 to −1.38); some combinations showed significant bleeding reduction vs PPI | All regimens had comparable safety profiles; H2RA showed non-significant trend toward higher bleeding risk (OR 3.77, 95%CI 0.94–15.18, P=0.062); **no regimen significantly reduced bleeding/perforation** |
| **7. Inconsistency assessment methods** | Global inconsistency via design-by-treatment Q tests; local inconsistency via node-splitting for closed loops | Global inconsistency via design-by-treatment interaction model; local inconsistency via node-splitting; **additional contribution matrix analysis** to quantify direct vs indirect evidence proportion |
| **8. Transitivity assessment approach** | **No transitivity assessment reported**; no discussion of effect modifier distribution across treatment groups | Explicit qualitative assessment of key effect modifiers (ulcer size, *H. pylori* status, lesion location, antithrombotic use); clearly stated limitation that quantitative assessment was not feasible due to incomplete reporting |
| **9. Certainty-of-evidence (GRADE) evaluation** | GRADE performed for primary outcomes; **most comparisons rated low or very low certainty** due to imprecision and indirectness; detailed results in supplementary material | GRADE performed for all pairwise comparisons across all 3 outcomes; P-CAB vs PPI and PPI+MP vs PPI rated **moderate certainty**; most other comparisons low/very low; complete GRADE table in main text (Table 5) |
| **10. Newly included trials in our analysis** | — | **11 additional RCTs** published after January 2025 or not included in the prior analysis: Kato2023, Kim2024, Shibata2025, Yang2025, plus 7 additional older RCTs covering P-CAB and H2RA monotherapy comparisons that were outside the scope of the prior study |
| **11. Principal findings** | PPI-based combinations likely accelerate ulcer healing vs PPI alone; bismuth magnesium granules + PPI optimal for 4-week healing, ecabet + PPI for 8-week healing, teprenone + PPI for bleeding prevention; conclusions described as hypothesis-generating and region-specific to East Asia | Time-dependent efficacy trends: P-CAB monotherapy numerically best for early (4-week) healing, PPI+MP ranked highest for late (8-week) healing; **no statistically significant inter-regimen differences** for any outcome; no regimen reduces delayed bleeding; findings limited by low-to-moderate GRADE certainty |
| **12.Explicit reasons for differences in conclusions** | ① Updated literature search identified 11 additional RCTs; ② broader intervention network includes P-CAB/H2RA regimens; ③ exclusion of animal experiments and conference abstracts; ④ different statistical framework (frequentist netmeta vs previous approach); ⑤ GRADE certainty was lower for most comparisons, leading to more conservative interpretation. | |
